# Impaired proteoglycan glycosylation, elevated TGF-β signaling, and abnormal osteoblast differentiation as the basis for bone fragility in a mouse model for gerodermia osteodysplastica

**DOI:** 10.1371/journal.pgen.1007242

**Published:** 2018-03-21

**Authors:** Wing Lee Chan, Magdalena Steiner, Tomasz Witkos, Johannes Egerer, Björn Busse, Shuji Mizumoto, Jan M. Pestka, Haikuo Zhang, Ingrid Hausser, Layal Abo Khayal, Claus-Eric Ott, Mateusz Kolanczyk, Bettina Willie, Thorsten Schinke, Chiara Paganini, Antonio Rossi, Kazuyuki Sugahara, Michael Amling, Petra Knaus, Danny Chan, Martin Lowe, Stefan Mundlos, Uwe Kornak

**Affiliations:** 1 Institut für Medizinische Genetik und Humangenetik, Charité –Universitätsmedizin Berlin, corporate member of Freie Universität Berlin, Humboldt-Universität zu Berlin, and Berlin Institute of Health, Berlin, Germany; 2 FG Development & Disease, Max-Planck-Institut fuer Molekulare Genetik, Berlin, Germany; 3 School of Biomedical Sciences, LKS Faculty of Medicine, The University of Hong Kong, Pok Fu Lam Road, Hong Kong; 4 Berlin School for Regenerative Therapies (BSRT), Charité –Universitätsmedizin Berlin, corporate member of Freie Universität Berlin, Humboldt-Universität zu Berlin, and Berlin Institute of Health, Berlin, Germany; 5 School of Biology, Faculty of Biology, Medicine and Health, University of Manchester, Manchester, United Kingdom; 6 Department of Osteology and Biomechanics, University Medical Center Hamburg-Eppendorf, Hamburg, Germany; 7 Lab. of Proteoglycan Signaling and Therapeutics, Faculty of Advanced Life Science, Graduate School of Life Science, Hokkaido University, Sapporo, Japan; 8 Institute of Pathology, University Clinic Heidelberg, Heidelberg, Germany; 9 Julius Wolff Institute, Charité –Universitätsmedizin Berlin, corporate member of Freie Universität Berlin, Humboldt-Universität zu Berlin, and Berlin Institute of Health, Berlin, Germany; 10 Department of Molecular Medicine, Unit of Biochemistry, University of Pavia, Pavia, Italy; 11 Institute for Chemistry and Biochemistry, Freie Universität, Berlin, Germany; 12 The University of Hong Kong—Shenzhen Institute of Research and Innovation (HKU- SIRI), Hi-Tech Industrial Park, Nanshan, Shenzhen, China; 13 Berlin-Brandenburg Center for Regenerative Therapies, Charité –Universitätsmedizin Berlin, corporate member of Freie Universität Berlin, Humboldt-Universität zu Berlin, and Berlin Institute of Health, Berlin, Germany; Stanford University School of Medicine, UNITED STATES

## Abstract

Gerodermia osteodysplastica (GO) is characterized by skin laxity and early-onset osteoporosis. *GORAB*, the responsible disease gene, encodes a small Golgi protein of poorly characterized function. To circumvent neonatal lethality of the *Gorab*^*Null*^ full knockout, *Gorab* was conditionally inactivated in mesenchymal progenitor cells (Prx1-cre), pre-osteoblasts (Runx2-cre), and late osteoblasts/osteocytes (Dmp1-cre), respectively. While in all three lines a reduction in trabecular bone density was evident, only *Gorab*^Prx1^ and *Gorab*^Runx2^ mutants showed dramatically thinned, porous cortical bone and spontaneous fractures. Collagen fibrils in the skin of *Gorab*^*Null*^ mutants and in bone of *Gorab*^Prx1^ mutants were disorganized, which was also seen in a bone biopsy from a GO patient. Measurement of glycosaminoglycan contents revealed a reduction of dermatan sulfate levels in skin and cartilage from *Gorab*^*Null*^ mutants. In bone from *Gorab*^Prx1^ mutants total glycosaminoglycan levels and the relative percentage of dermatan sulfate were both strongly diminished. Accordingly, the proteoglycans biglycan and decorin showed reduced glycanation. Also in cultured *GORAB*-deficient fibroblasts reduced decorin glycanation was evident. The Golgi compartment of these cells showed an accumulation of decorin, but reduced signals for dermatan sulfate. Moreover, we found elevated activation of TGF-β in *Gorab*^Prx1^ bone tissue leading to enhanced downstream signalling, which was reproduced in *GORAB*-deficient fibroblasts. Our data suggest that the loss of *Gorab* primarily perturbs pre-osteoblasts. GO may be regarded as a congenital disorder of glycosylation affecting proteoglycan synthesis due to delayed transport and impaired posttranslational modification in the Golgi compartment.

## Author summary

Gerodermia osteodysplastica (GO) is segmental progeroid disorder affecting connective tissues and bone, leading to extreme bone fragility. The cause are loss-of-function mutations in the Golgi protein GORAB, whose function has been only partially unravelled. Using several mouse models and patient-derived primary cells we elucidate that loss of Gorab elicits a defect in proteoglycan glycanation, which is associated with collagen disorganization in dermis and bone. We also found evidence for TGF-β upregulation and enhanced downstream signalling. If these changes occur in mesenchymal stem cells or early osteoblasts they impair osteoblast differentiation resulting in cortical thinning and spontaneous fractures. We thus match GO mechanistically with also phenotypically overlapping progeroid connective tissue disorders with glycanation defects.

## Introduction

Bone mass is highly heritable and largely determined by bone growth during childhood and adolescence leading to the so-called peak bone mass, and the rate of subsequent bone loss at older ages [1]. Gerodermia osteodysplastica (GO; OMIM #231070) belongs to the group of autosomal recessive cutis laxa (ARCL) syndromes characterized by lax, wrinkled skin, a generalized connective tissue weakness, and a progeroid appearance [2–4]. GO features pronounced osteoporosis leading to pathological fractures already in childhood. GORAB, the gene product defective in GO, is a coiled-coil containing peripheral membrane protein that is recruited to the Golgi compartment via a specific, GTP-dependent interaction with the small GTPases ARF5 and RAB6 [5]. Due to this fact, GORAB has been suggested to belong to the group of golgins, small GTPase effector proteins involved in different steps of Golgi-related transport processes. Nevertheless, the physiological role or GORAB in development and homeostasis of the skeleton and of connective tissues is not well understood.

The Golgi compartment is a central hub for protein trafficking and posttranslational modification within the secretory pathway, among which glycosylation processes are most prominent [6]. While the classical disorders of glycosylation (CDGs) affect N-glycosylation leading to a prototypical combination of neurological, hepatic, and gastrointestinal symptoms, impairment of the different types of O-glycosylation often causes musculoskeletal phenotypes [7]. Glycosaminoglycans (GAGs), mostly attached to proteoglycan core proteins through glycanation processes in the Golgi apparatus, importantly contribute not only to tissue elasticity and organization of the ECM, but also regulate growth factor signaling [6]. One example are the small leucine rich proteoglycans decorin and biglycan, which carry dermatan or chondroitin sulfate GAG chains and regulate collagen fibrillogenesis and signaling mediated by diverse ligands [8]. Several known disorders of GAG synthesis are characterized by a prematurely aged appearance and fragile bones [9–11]. However, the molecular background of these pathologies has only partially been unraveled.

We here report on the characterization of different constitutive and conditional mouse models indicating that GO is due to osteoblast dysfunction and that *Gorab* is most relevant in early stages of osteoblast differentiation. *Gorab* inactivation reduces dermatan sulfate levels and proteoglycan glycanation in skin and bone tissue. Loss of GORAB in fibroblasts leads to decorin retention and lower GAG levels in the Golgi compartment. Altered proteoglycans are not only associated with disorganization of the collagen matrix, but also with aberrant TGF-β activation, which likely perturbs differentiation and function of osteoblast lineage cells.

## Results

### Inactivation of *Gorab* in mesenchymal stem cells or pre-osteoblasts causes osteopenia and thinning of cortical bone

**Fig 1:**
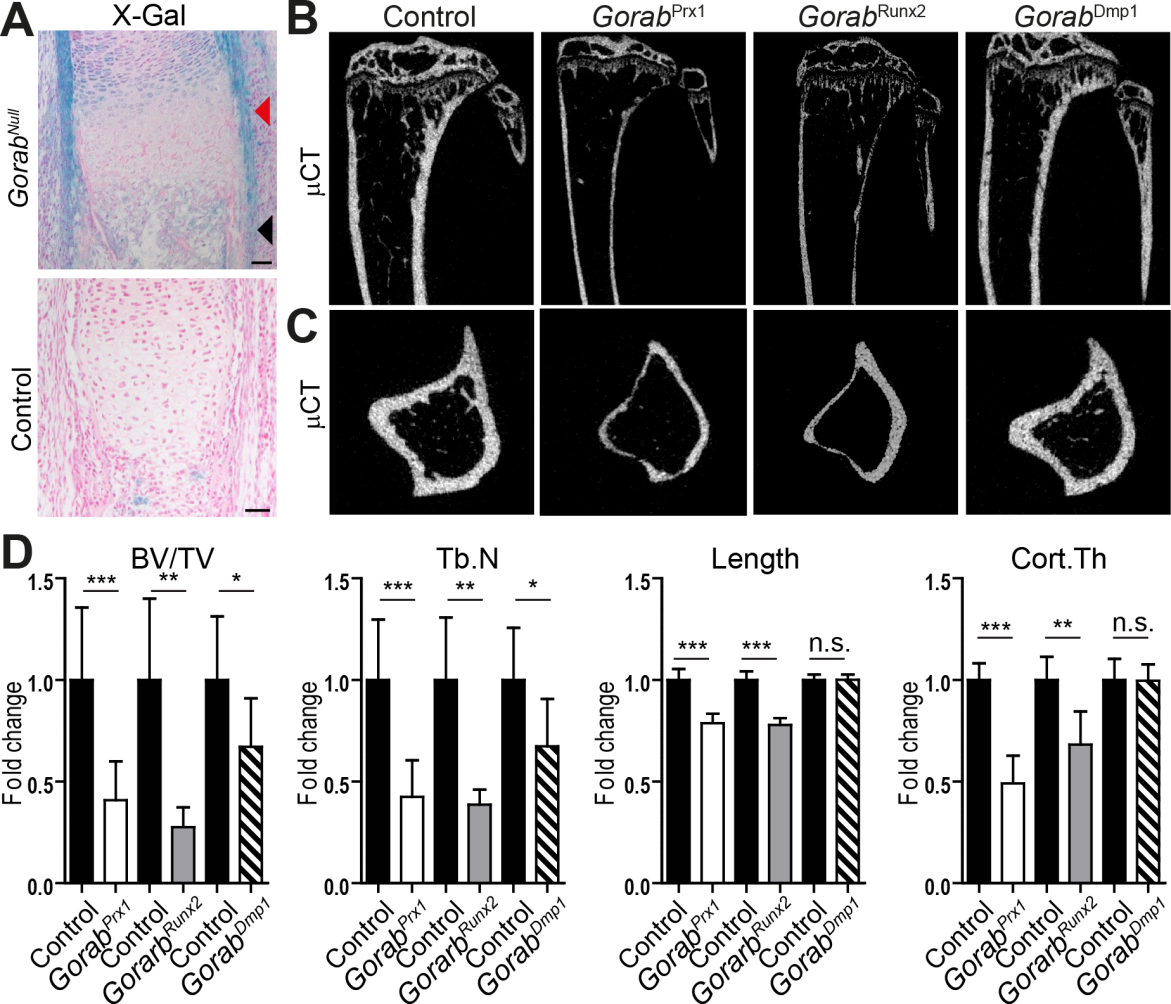
*Gorab* conditional inactivation in mesenchymal progenitors and pre-osteoblasts, but not at later stages leads to severe cortical bone thinning. (A) *Gorab* expression in chondrocytes, perichondrium (red arrowhead), and periosteum (black arrowhead) as indicated by LacZ staining of E18.5 *Gorab*^*Null*^ tibia. Scale bar = 50μm. (B) Sagittal view and (C) transverse view of reconstructed μCT image of tibia from twelve week old *Gorab*^Prx1^, *Gorab*^Runx2^, *Gorab*^Dmp1^ and control animals. (D) Quantitative μCT analysis given as fold changes for trabecular bone volume (BV/TV), length and cortical thickness (Cort.Th), of tibia of twelve week old *Gorab*^Prx1^ (N = 15), *Gorab*^Runx2^ (N = 5), *Gorab*^Dmp1^ (N = 8) and littermate control animals, N = 14, N = 5 and N = 9 for each individual conditional mouse line, respectively. Values were normalized to the control littermates of each mutant line to facilitate comparison.

The majority of *GORAB* mutations found in GO patients lead to a loss of the GORAB protein [3]. Therefore, we first constitutively inactivated *Gorab* in a genetrap mouse line and in a complete knockout after removing the floxed exons 2 and 3 from the *Gorab*^flox^ locus in the germline to study the unknown cause for bone fragility in GO (**S1A–S1D Fig**). Both mouse lines, which are in the following referred to as *Gorab*^Null^, showed an identical phenotype with absence of skin changes reminiscent of cutis laxa, but early lethality due to respiratory distress, most likely secondary to decreased alveolar airspace (**S2A–S2D Fig**). This is in line with an independent description of *Gorab*^Null^ mice [12]. Apart from enlarged fontanels no significant skeletal abnormalities were identified (**S3A–S3E Fig**). Especially the cortical bone porosity was in the normal range for this developmental stage (**S3C and S3D Fig**). We took advantage of the β-galactosidase expressed from the *Gorab* genetrap locus to visualize the expression pattern in the developing skeleton since available Gorab antibodies lack specificity in immunohistology (**Fig 1A**). Strongest signals were seen in the perichondrium and the periosteum, which governs formation of the cortical bone. Furthermore, we assessed *Gorab* expression in comparison to several marker genes in calvarial osteoblasts differentiated *in vitro* for 12 days until *Dmp1*, a marker for late osteoblasts/osteocytes, was robustly expressed (**S4A Fig**). *Gorab* expression peaked at day 6 of differentiation, together with type 1 collagen (*Col1a1*) and decorin (*DCN*), which was induced about 40-fold compared to day 0 (**S4A Fig**). We also differentiated osteoblast precursors isolated from E18.5 and P0 *Gorab*^Null^ calvariae into mineralizing osteoblasts *in vitro*. Neither alkaline phosphatase activity nor mineralization as measured by alizarin red showed any differences correlating with the dramatic bone changes found in GO (**S3G and S3H Fig**). We therefore hypothesized that development of the bone phenotype in our murine GO model occurs mainly postnatally when the embryonic woven bone is converted into mature lamellar bone, a process which is not faithfully recapitulated in the usual 2D osteoblast cultures.

To investigate the role of *Gorab* in postnatal skeletal development we generated conditional *Gorab*^flox^ mice to selectively prevent *Gorab* expression in bone tissue while preserving expression in the lung (**S1C and S1D Fig**). We inactivated the gene in the limb bud mesenchyme, pre-osteoblasts, and late osteoblasts/osteocytes by crossing *Gorab*^flox^ mice with Prx1-, Runx2-, and Dmp1-cre mice, respectively, to investigate at which differentiation stage osteoblast lineage cells are most sensitive to a loss of *Gorab* (**S4A Fig**) [13–15]. *Gorab* expression in cortical bone was reduced to a similar degree in all three conditional mouse lines (**S4B Fig**). The resulting *Gorab*^Prx1^ and *Gorab*^Runx2^ mutants showed a retardation of long bone growth and a dramatic loss of cortical and trabecular bone (**Fig 1B–1D**). In contrast, only a mild loss of trabecular bone was evident in tibiae from *Gorab*^Dmp1^ mutants (**Fig 1B–1D**) (**S5A Fig**). Since *Prx1* is not expressed in the axial skeleton no changes were observed in *Gorab*^Prx1^ vertebral bone structure, but *Gorab*^Runx2^ and *Gorab*^Dmp1^ mutants showed a clear vertebral osteopenia (**S5B Fig**). These data suggest that, at least for cortical bone development, *Gorab* function is most important during differentiation of pre-osteoblasts from mesenchymal progenitor cells.

### Cortical thinning and porosities result in spontaneous fractures in *Gorab*^Prx1^ mutants

**Fig 2:**
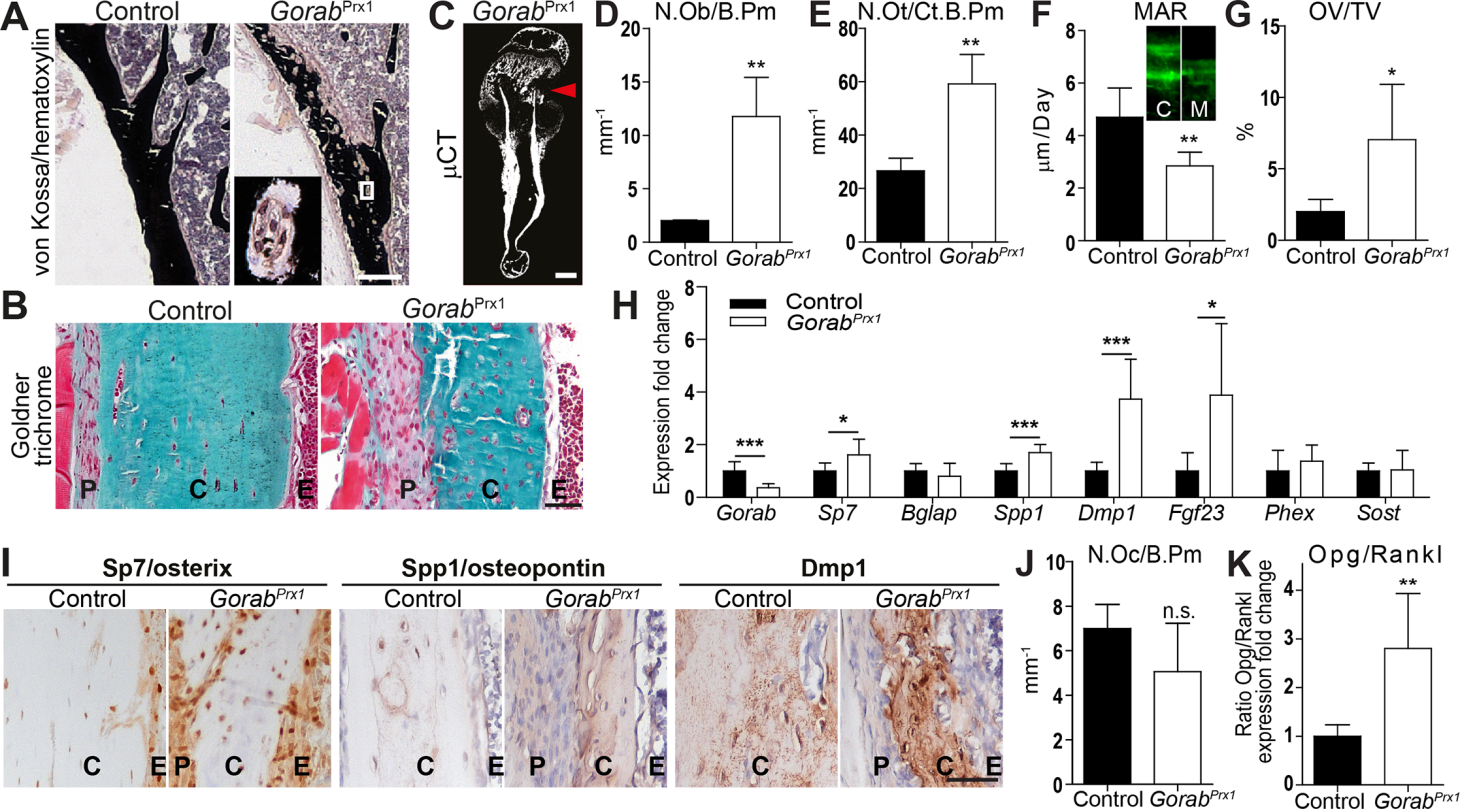
Cortical porosity and osteoblast dysfunction in the *Gorab*^Prx1^ model recapitulate the gerodermia osteodysplastica bone phenotype. (A) Von Kossa / hematoxylin stained cortical bone of twelve week old *Gorab*^Prx1^ mouse. Inset showing magnified view of large pores in the mutant diaphysis. Scale bar = 200 μm. (B) Sections of metaphyseal cortical bone of the tibia of four week old *Gorab*^Prx1^ mice stained with Masson Goldner trichrome. Scale bar = 100μm. (C) μCT reconstructed image of a spontaneously fractured humerus from a four week old *Gorab*^Prx1^ mouse. Arrowhead = fracture site. Scale bar = 1mm. (D) Histomorphometric quantitation number of osteoblast per bone perimeter (N.Ob/B.Pm), (E) number of osteocytes per cortical bone perimeter (N.Ot/cort B.Pm). (F) Mineral apposition rate (MAR) at the endosteum of tibia midshaft in four week old *Gorab*^*Prx1*^ (N = 4). Inset showing calcein double labeling, C = control, M = mutant. (G) osteoid volume (OV/TV) in secondary spongiosa of the proximal tibia in *Gorab*^Prx1^ mutants vs. controls at four weeks of age (N = 4–6). (H) Altered expression of osteoblast lineage marker genes in femoral cortical bone of four week old *Gorab*^Prx1^ mutants (N = 6–8). (I) Immunohistochemical detection of Sp7/osterix, Spp1/osteopontin and Dmp1 expression in cortical bone. Note higher number of osterix + cells in *Gorab*^Prx1^ mutants. Scale bar = 50μm. (J) Number of osteoclasts per bone perimeter (N.Oc/B.Pm). (K) Opg to Rankl expression ratio in four week old *Gorab*^*Prx1*^ mutants (N = 6). P = periosteum, C = cortical bone, E = endosteum.

We then focused our analysis on the *Gorab*^Prx1^ model, which corresponded well to the GO long bone phenotype. The biomechanical and material properties of bone tissue from this mouse model have been described elsewhere [16]. Histological analysis of different stages of postnatal development revealed that the bone anomalies were most striking in the tibia at four weeks of age. Thinning and porosities of the cortical bone were most pronounced in the posterior metaphyses (**Fig 2A**). Several cells were observed in the large cortical pores suggesting that they represent merged osteocyte lacunae. The overall number of osteocyte lacunae was elevated and the periosteum was strongly thickened at the expense of cortical bone (**Fig 2B**). The metaphyseal cortical bone changes in *Gorab*^Prx1^ culminated in very high bone fragility as mirrored by spontaneous fractures in up to 80% homozygous mutants within the first four weeks of postnatal development (**Fig 2C**). Humeri and tibiae were most frequently affected, often leading to deformations. We postulate that *Gorab* deficiency in early osteoblast lineage cells during the first weeks of postnatal development cannot be compensated due to the immense rate of ECM production, which is known to exert stress on the cells secreting ECM components.

### Altered differentiation and gene expression of osteoblast lineage cells in *Gorab*^Prx1^ mutants

We therefore next wanted to know more about the impact of loss of *Gorab* function on the cells of the bone multicellular unit. Histomorphometric analysis of *Gorab*^Prx1^ tibiae revealed higher osteoblast and osteocyte numbers (**Fig 2D and 2E**). In spite of more abundant osteoblasts, mineral apposition rate was reduced in *Gorab*^Prx1^ mutants and an increased osteoid surface indicated impaired mineralization (**Fig 2F and 2G**). A reduced cortical bone mineral to matrix ratio was also detected in twelve week old *Gorab*^Prx1^ mutants by Fourier transform infrared (FTIR) imaging [16]. These findings were confirmed in a bone biopsy from a GO patient, demonstrating that our mouse model closely recapitulates the human condition (**S6 Fig**). To elucidate the basis for these osteoblast lineage anomalies we performed expression analyses in tibial cortical bone tissue. Individual qPCR assessment of osteoblast marker genes revealed an upregulation of *Spp1* and *Sp7* (**Fig 2H**). In addition, genome-wide expression analysis of *Gorab*^*Prx1*^ diaphyseal cortical bone by array hybridization and qPCR verification showed upregulation of the osteocyte marker genes *Dmp1* and *Fgf23*, (**Fig 2H**)(**S1 Table**). The upregulation of *Dmp1* was already evident in E18.5 *Gorab*^Null^ bones, together with a slight suppression of *Sost*, which could indicate a delay in osteocyte maturation during prenatal development (**S3F Fig**). Immunohistochemistry confirmed increased levels of the proteins osteopontin, osterix, and dentin matrix protein 1 in *Gorab*^*Prx1*^ cortical bone (**Fig 2I**). Interestingly, also the genes *Ank*, *Enpp1*, and *Mepe* known to inhibit mineralization were induced in *Gorab*^*Prx1*^ mutants (**S1 Table**), possibly contributing to the observed osteoid mineralization defect (**Fig 2G**). With the exception of normal *Sost* expression levels the gene expression profile in *Gorab*^*Prx1*^ mutant cortical bone resembled that of osteoid osteocytes indicating that terminal osteocyte differentiation was impaired as a consequence of *Gorab* inactivation in osteoblast precursors [17].

Osteoclast numbers were not significantly changed in *Gorab*^*Prx1*^ trabecular bone (**Fig 2J**). Cell counts in the available human bone biopsy neither did show significantly elevated osteoclast numbers (**S6 Fig**). Although ECM disorganization often induces increased osteoclast numbers and activity, this is probably suppressed in *Gorab*^*Prx1*^ mutants by an elevated Opg/Rankl ratio detected by expression analysis (**Fig 2K**) [18, 19]. Taken together, these data suggest low turnover kinetics due to functional impairment of the osteoblast lineage. This is in contrast to osteogenesis imperfecta (OI), the most common type of congenital bone disease with fractures, which is characterized by high turnover kinetics [18].

### Abnormal collagen fibrillogenesis in *Gorab* deficient skin and bone tissue

**Fig 3:**
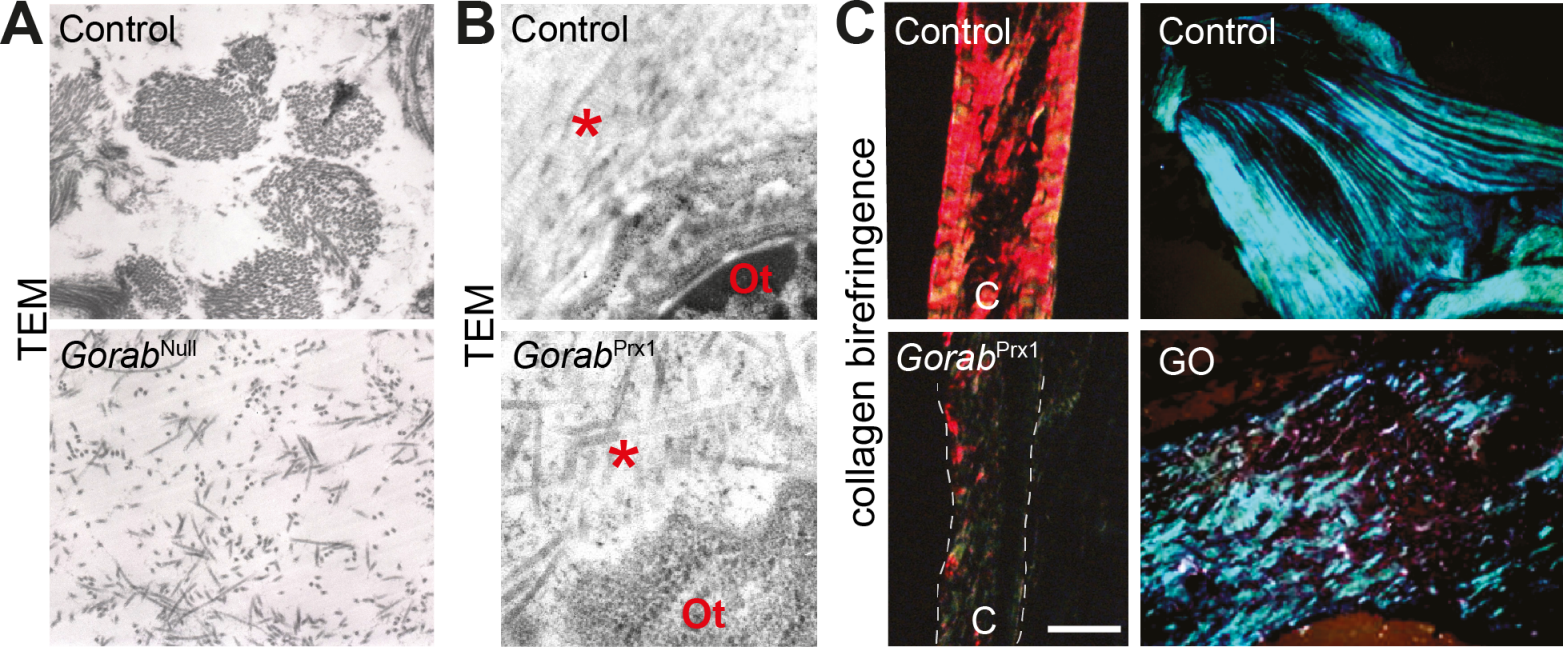
Disrupted collagen network in skin and bone tissue deficient for *Gorab*. (A) Electron microscopy of skin from E18.5 *Gorab*^Null^ mouse (magnification = 11700x) and (B) bone from tibia of eight week old *Gorab*^Prx1^ mouse. Asterisk marks collagen fibers, Ot = osteocyte. (C) Collagen fiber orientation in cortical bone of four week old *Gorab*^Prx1^ mutants and in a GO patient bone biopsy stained with picrosirius red or toluidine blue, respectively, and imaged under polarized light. Note fragmentation of birefringence signals. For each experiment at least N = 3 mice were analyzed, representative images are shown.

Gerodermia osteodysplastica is characterized by a congenital ligamentous laxity indicating collagen abnormalities, and dermal elastic fiber changes. Investigating the dermis of newborn *Gorab*^Null^ mice by electron microscopy we found a disorganization of collagen fibers (**Fig 3A**). In contrast to control skin almost no fibril formation was seen. Elastic fibers are not yet formed at this developmental stage and could therefore not be studied. *Gorab*^*Prx1*^ cortical bone was thinner and showed reduced stiffness and increased fragility (**Fig 2B**)[16]. The mechanical properties of bone are also determined by collagen organization. Similar to the skin we also found missing alignment of collagen fibers surrounding the osteocytes in four week old *Gorab*^*Prx1*^ mutants (**Fig 3B**). Picrosirius red staining and polarized light microscopy confirmed globally impaired collagen fibrillogenesis in tibial cortical bone from *Gorab*^*Prx1*^ mutants and in a pelvic bone biopsy from a nine year old GO patient (**Fig 3C**). Our results show the presence of impaired collagen fibrillogenesis in GO, at least during development of bone and connective tissues.

### Reduced dermatan sulfate content and impaired proteoglycan glycanation in *Gorab* deficient skin and bone tissue

**Fig 4:**
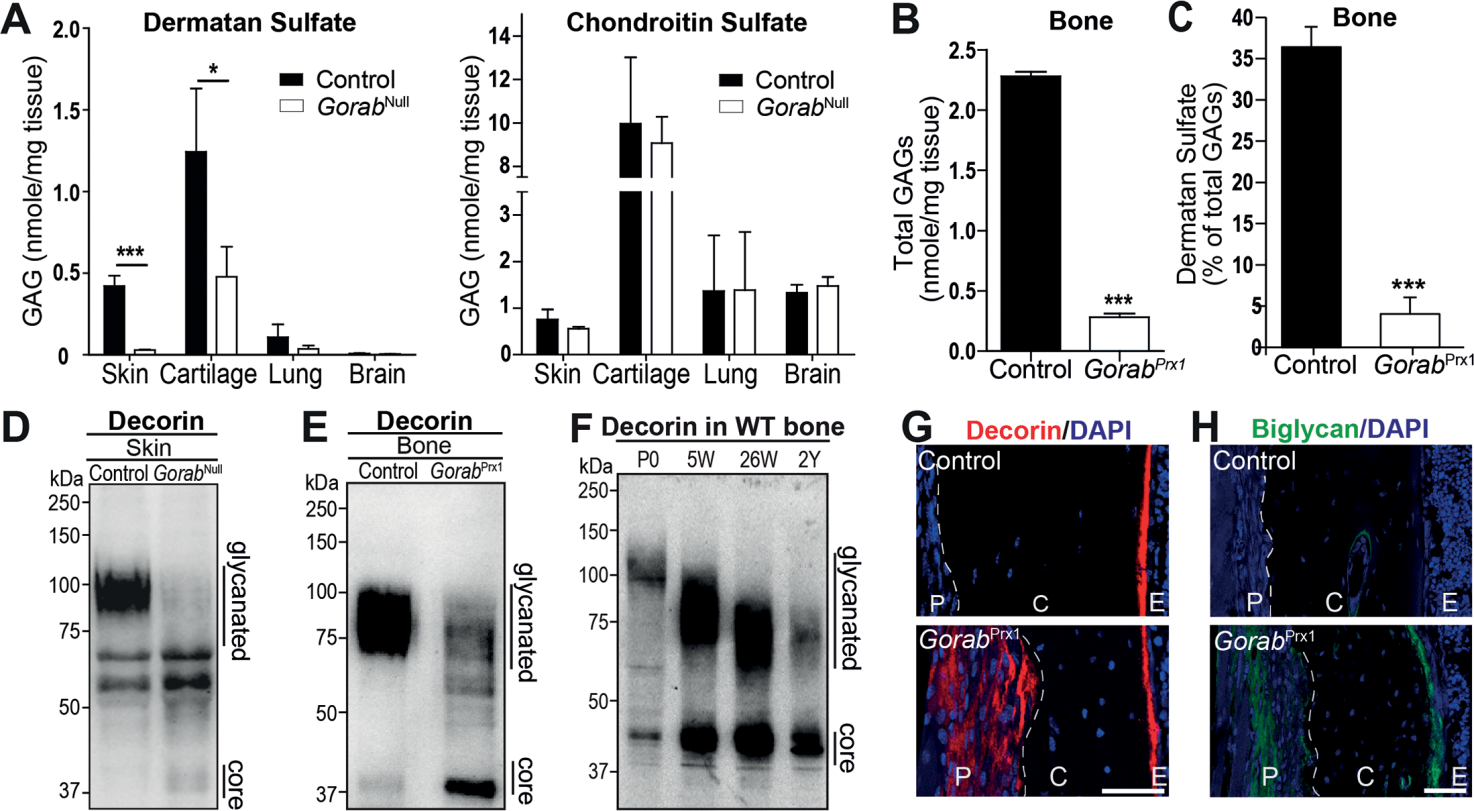
Loss of *Gorab* resulted in underglycanation of proteoglycans. (A) Quantitation of dermatan sulfate and chondroitin sulfate in skin, cartilage, and lung samples from E18.5 *Gorab*^Null^ mice (N = 3–4). (B) Total amount of GAGs in the cortical bone of femora from four week old *Gorab*^*Prx1*^ mutants and littermate controls (N = 3–4). (C) Percentage of dermatan sulfate in the total amount of glycosaminoglycans (GAGs). (D) Immunoblotting for decorin in skin samples from E18.5 *Gorab*^Null^ mice. Loss of the 100kDa band, corresponding to the fully glycanated decorin, and higher intensity the core protein band in mutant lysate indicate a glycanation defect. (E) Immunoblotting for decorin in cortical bone lysates from four week old *Gorab*^Prx1^ mice and littermate controls also showing higher intensity of lower bands in mutant. (F) Immunoblot of decorin in cortical bone lysates from wildtype (WT) mice at different ages: newborn (P0), 5 weeks (5W), 26 weeks (26W) and 2 years (2Y). Note reduction in glycanation with increasing age. (G) Immunofluorescence detection of decorin and (H) biglycan in tibia of four week old *Gorab*^Prx1^ mice. Sections were not pretreated with chondroitinase. Higher staining intensities therefore indicate lower glycanation of the core proteins in the periosteum. P = periosteum, C = cortical bone. Scale bar = 50μm. Experiments (D) to (H) were repeated at least three times with independent biological samples, representative results are shown.

The Golgi localization of GORAB is suggestive of a function in protein secretion and/or modification. Metabolic labeling of fibroblasts from GO patients showed no influence of GORAB on global protein or collagen secretion (**S7A and S7B Fig**). However, collagen and elastic fiber changes similar to GO were described in other progeroid connective tissue disorders secondary to impaired glycanation of proteoglycans [9–11]. To test a possible involvement of proteoglycans in GO pathology, we measured glycosaminoglycans (GAGs) in tissues from E18.5 *Gorab*^Null^ embryos. We observed a significant reduction in the amount of dermatan sulfate, but not of other GAGs in skin and cartilage, indicating a specific defect (**Fig 4A**)(**S7C Fig**). In *Gorab*^*Prx1*^ tibial cortical bone the total GAG levels as well as the relative amounts of dermatan sulfate levels were strongly reduced (**Fig 4B and 4C**). Biglycan and decorin are major dermatan- and chondroitin sulfate-carrying proteoglycans in bone and connective tissues. Their fully glycanated form can be difficult to detect in immunoblots with tissue lysates due to their apparent size (decorin 100 kDa, biglycan >200 kDa) and low accessibility of the epitope residing in the core proteins that have a size of 42 kDa and 45 kDa, respectively. Immunoblot detection of both proteoglycans in skin tissue lysates showed a partial loss of the fully glycanated forms and stronger core protein bands in *Gorab*^Null^ mutants suggesting absent/shortened GAG chains (**Fig 4D**)(**S7D Fig**). Furthermore, after treatment with chondroitinase ABC the core proteins of decorin and biglycan both gave stronger signals indicating lesser glycanation (**S7E and S7F Fig**). No upregulation of mRNA expression was detected, which could alternatively explain the stronger core protein bands (**S7G and S7H Fig**). Also in bone lysates from four week old *Gorab*^*Prx1*^ animals the fully glycanated form of decorin was less abundant compared to littermate controls and bands with lower apparent weight were enhanced (**Fig 4E**). Reduced decorin glycanation has been demonstrated to be a consequence of aging in human skin [20]. We found a similar reduction in decorin glycanation with aging in cortical bone from wildtype mice (**Fig 4F**). Decorin glycanation in 4 week old *Gorab*^*Prx1*^ bone was reduced to a level similar to that of 26 week old controls (compare **Fig 4E and 4F**). Taken together, loss of *Gorab* caused a general reduction of proteoglycan glycanation, exemplified by the changes in glycanation status of decorin and biglycan, similar to that found in the aged ECM.

Decorin and biglycan play a vital role in collagen fibrillogenesis [21], are highly expressed in osteoblasts (**S4A Fig**) and were linked to alterations of matrix mineralization, growth factor signaling, and bone fragility [22–25]. In immunohistology we found enhanced signals for biglycan and decorin in the ECM of the thickened periosteum in *Gorab*^*Prx1*^ tibiae, most likely due to enhanced accessibility of the epitope as a consequence of lower glycanation (**Fig 4G and 4H**). This suggested a role of these pathologically glycanated proteoglycans in the abnormal gain of periosteum thickness.

### *GORAB* deficiency in fibroblasts leads to reduced glycanation and accumulation of decorin in the Golgi compartment

**Fig 5:**
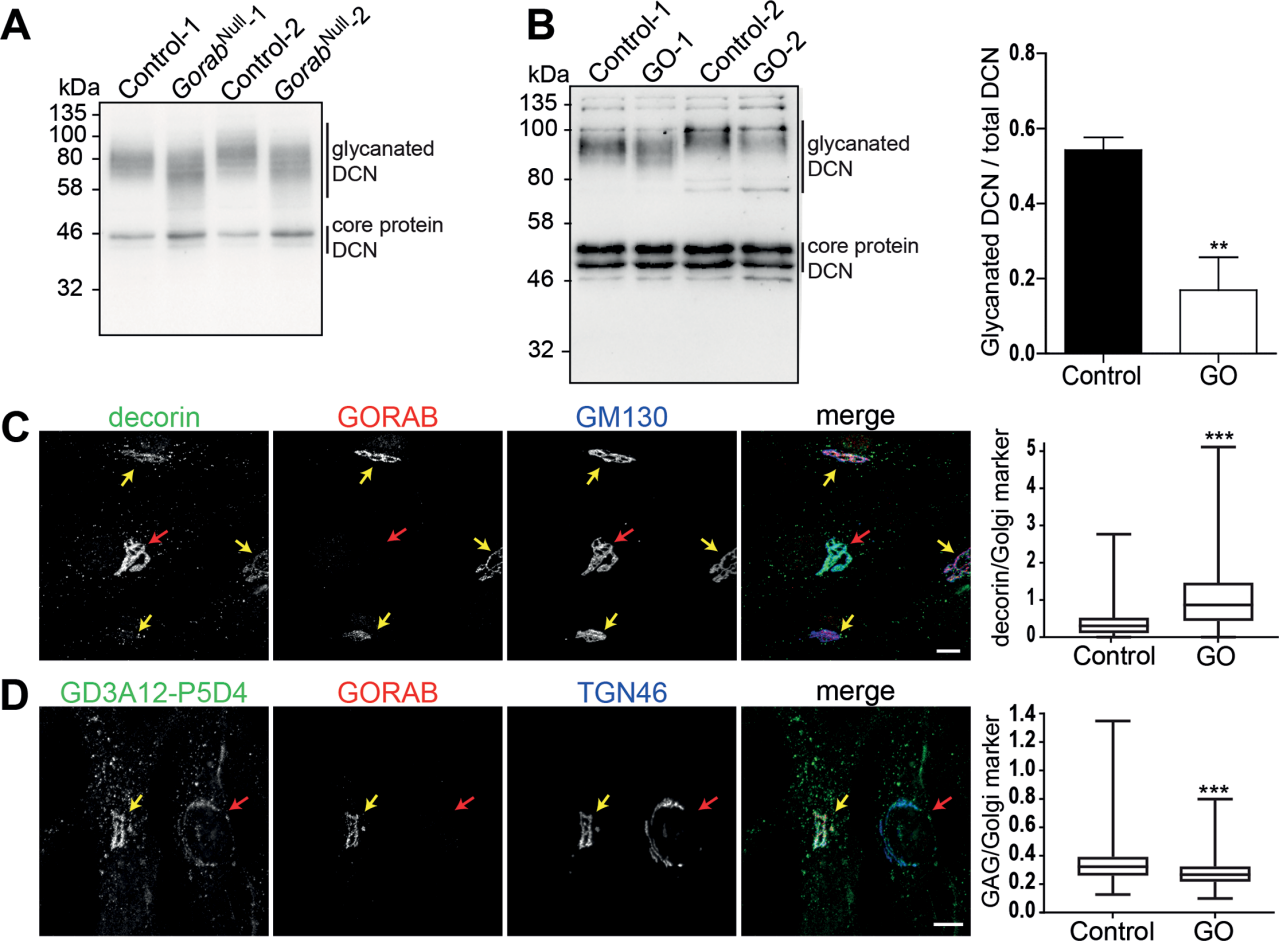
Golgi retention and reduced glycanation of decorin in *Gorab*-deficient fibroblasts. (A) Western blot analysis of decorin in control and *Gorab*^Null^ MEF cell lysates. The size ranges for the fully glycanated form of decorin (DCN) and of its core protein are indicated. (B) Left, western blot analysis of decorin in lysates of the extracellular matrix produced by cultured control and GO fibroblasts. Right, levels of glycanated decorin were quantified against total decorin levels (N = 3). (C) Analysis of intra-Golgi levels of decorin in co-cultured control and GO human skin fibroblasts. Immunofluorescence labeling was performed with anti-decorin, anti-GORAB and anti-GM130 antibodies. GORAB staining was used to distinguish control (yellow arrows) and GO (red arrows) cells. Decorin fluorescence intensity in both cell types was normalized against that of the Golgi marker GM130 (N = 3, >500 cells analyzed per cell line). Scale bar = 10 μm. (D) Analysis of intra-Golgi levels of dermatan sulfate (DS)-modified proteins in co-cultured control (yellow arrows) and GO (red arrows) human skin fibroblasts. Cells were labeled with anti-DS (GD3A12), anti-GORAB and anti-TGN46 antibodies. The intensity of the GD3A12 fluorescence signals were measured relative to that of the Golgi marker TGN46 (N = 3, >500 cells analyzed per cell line). Scale bar = 10 μm.

In order to investigate whether the observed proteoglycan abnormalities are due to a perturbation of the producing cells we investigated decorin production in cultured fibroblasts. In lysates from confluent *Gorab*^*Null*^ and control fibroblasts we observed a downward shift of the band corresponding to the glycanated form (**Fig 5A**). A similar finding was obtained for biglycan (**S7E Fig**). Also decorin secreted by fibroblasts from GO patients displayed a significant reduction of the fully glycanated form (**Fig 5B**). These data demonstrate that the reduced glycanation of proteoglycans in *Gorab*-deficient mouse mutants can be recapitulated *in vitro* in both mouse and human fibroblast cell lines.

The GORAB protein is associated with the medial/trans Golgi compartment, where it supposedly regulates transport processes [5]. Therefore, we hypothesized that the decorin abnormalities could be due to impaired intracellular trafficking. In order to prevent influence of extrinsic factors and to ensure comparable immunofluorescence stainings, the following experiments were done in co-cultures of fibroblasts derived from control and GO individuals. Both cell types could be distinguished by presence or absence of GORAB immunofluorescence staining (**Fig 5C**). GORAB-deficient cells showed increased co-localization of decorin and the Golgi marker GM130 in immunofluorescence compared to GORAB-expressing control cells (**Fig 5C**). We also assessed the dermatan sulfate levels at the Golgi compartment detected by antibody GD3A12 through immunofluorescence co-staining with the Golgi marker TGN46 [26]. Although decorin accumulated at the Golgi compartment, the signals for dermatan sulfate chains were less intense (**Fig 5D**). Thus, loss of GORAB seems not only to reduce anterograde trafficking of decorin within the Golgi, but also dermatan sulfate attachment to its core protein. Given the abovementioned changes in total GAG levels in bone and in biglycan glycanation (**S7F Fig**), this effect does not seem to be limited to decorin, but probably affects proteoglycans in general.

### Enhanced TGF-β activation and downstream signaling

**Fig 6:**
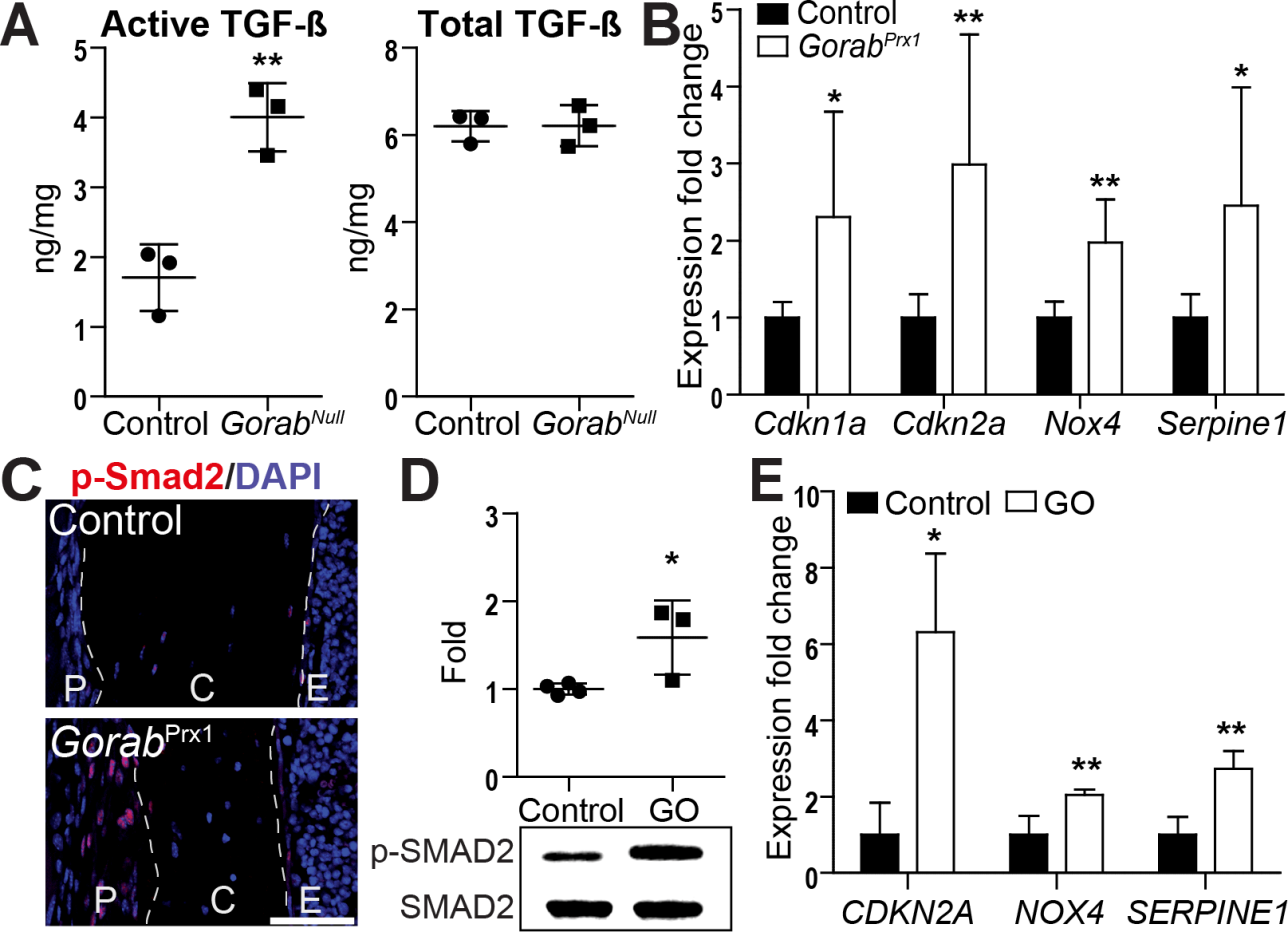
Elevated TGF-β signaling in *Gorab*-deficient skin, bone, and fibroblasts. (A) Quantitation of active and total TGF-β in skin lysates from E18.5 *Gorab*^Null^ mice (N = 3). (B) Enhanced expression of TGF-β regulated genes in the diaphysis of four week old *Gorab*^Prx1^ mutants (N = 7–8). (C) Immunofluorescence staining for p-SMAD2 in *Gorab*^Prx1^ mutants and controls at four weeks of age. Representative picture of N = 4 per group. Note stronger signals in periosteum in mutants. Scale bar = 50μm. (D) Western blot of p-SMAD2 in confluent fibroblasts from GO patients and healthy controls (N = 3) and quantitative evaluation. (E) Quantitative PCR to measure expression of TGF-β responsive genes in GO patient-derived fibroblasts (N = 4). P = periosteum, C = cortical bone. E = endosteum.

Besides collagen, decorin and biglycan also bind TGF-β with high affinity and modulate its bioavailability and interaction with receptors [27, 28]. TGF-β is a central regulator of bone remodeling produced by osteoblasts. It is secreted predominantly in its latent, inactive form and deposited into the matrix from where it gets activated by osteoclast activity and proteolytic cleavage [29, 30]. Excessive TGF-β signaling has been shown to be crucial in the pathology of osteogenesis imperfecta and several connective tissue disorders [19, 31]. Using a TGF-β reporter cell line, we found an increase of active TGF-β in *Gorab*^Null^ skin lysates, while total TGF-β levels remained constant (**Fig 6A**). Evidence for elevated TGF-β signaling was also indicated by the upregulation of TGF-β responsive genes such as *Serpine1* in cortical bone of *Gorab*^Prx1^ mutants (**Fig 6B**). Increased nuclear staining of p-Smad2 was found in *Gorab*^Prx1^ bone tissue, in particular in cells located in the enlarged periosteum (**Fig 6C**), indicating activation of TGF-β signaling in osteoblast lineage cells. In low passage (5–10) GO skin fibroblasts we also observed increased levels of p-SMAD2, and elevated expression of TGF-β responsive genes (**Fig 6D and 6E**). These findings suggest that GO can be regarded as a congenital disorder of glycosylation (CDG) leading to a disorganized collagen network and TGF-β activation due to abnormal function of the underglycanated proteoglycans, including decorin and biglycan.

## Discussion

Our results suggest that the loss of Gorab in the Golgi compartment causes reduced proteoglycan glycanation, abnormalities in collagen networks, and subsequent TGF-β overactivation. GAGs are essential for the function of proteoglycans. This is highlighted by several congenital disorders of glycosylation (CDGs) caused by deficient attachment of GAGs to the proteoglycan core protein in the Golgi compartment [9–11]. Besides generalized connective tissue problems and a progeroid appearance some of these disorders also show a dramatic bone fragility. In contrast to GO, these disorders are readily classified as CDGs since they affect enzymes involved in GAG synthesis. The typical readout used in functional studies for these disorders is decorin glycanation status [32]. Our data imply a strong clinical and biochemical overlap of these disorders with GO and suggest that GO might be regarded as a CDG.

We used different cre-expressing mouse lines to learn more about the stage of osteoblast differentiation at which *Gorab* is most essential. *Gorab* expression peaks in mature osteoblasts, together with ECM proteins like type 1 collagen. It seems that only a loss of *Gorab* expression before this osteoblast differentiation stage in *Gorab*^Prx1^ and *Gorab*^Runx2^ mice gives rise to the full phenotypic picture, while deletion in late osteoblasts in the *Gorab*^Dmp1^ line has milder effects. Late osteoblasts are characterized by reduced ECM production and start to convert into osteocytes [17]. Likewise, the *Gorab*^Prx1^ and *Gorab*^Runx2^ mouse models develop age-related phenomena like cortical porosity and low bone turnover already during bone growth, thus hampering bone mass accrual. We speculate that the reason for this might be the strong ECM production during the postnatal growth spurt. From these aspects we conclude that loss of GORAB primarily perturbs strongly ECM secreting cells, which might explain the selective affection of bone and connective tissues in GO in spite of the ubiquitous expression of *GORAB*.

The small leucine-rich proteoglycans (SLRPs) are important for ECM homeostasis by regulating collagen fibrillogenesis and binding to TGF-β and other ligands and receptors in a complex manner [8]. It has been hypothesized that upon alteration of the collagen network SLRP binding to the ECM is loosened leading to increased release of active TGF-β [19]. Diffusible biglycan and decorin might even enhance binding of TGF-β to its receptor, thereby further inducing collagen production to repair the defective collagen network [33]. The knockout of decorin and also the exchange of its GAG-carrying serine residue have only mild phenotypic effects [34, 35]. In contrast, loss of biglycan leads to osteopenia and cortical thinning quite similar to *Gorab*^Prx1^ and *Gorab*^Runx2^ mutants [25]. Human biglycan loss-of-function mutations cause aortic dilatation, malar hypoplasia, and osteopenia with thin cortices, a clinical picture somewhat reminiscent of GO [36]. Defective decorin glycanation, which we show in aging bone tissue, has also been demonstrated to be a consequence of skin aging [20]. Generally, the sulfated GAG content of many tissues is reduced with increasing age [37]. From an evolutionary standpoint it was suggested that age-related changes in glycosylation patterns might shield the organism from cancer by lowering proliferation signals and enhancing differentiation signals like TGF-β [38]. Binding of biglycan and decorin to TGF-β and to type 1 collagen is mediated by the core protein and can be lowered by the attached GAG chains [39]. On the other hand, dermatan sulfate was shown to bind to collagen fibrils in a reproducible pattern, which might explain the observed differences in the binding patterns of glycanated and non-glycanated decorin to collagen fibrils [40, 41]. We therefore assume that reduced glycanation of SLRPs plays a leading role in the pathogenesis of GO.

In our mouse models and also in cultured cells we found evidence for elevated TGF-β activation, which we propose to be a consequence of the disrupted ECM. TGF-β signaling plays pivotal role in bone homeostasis [30]. TGF-β stored in the bone matrix is released and activated during bone resorption. This is turn attracts bone osteoprogenitor cells to the resorption sites, which subsequently proliferate and differentiate into osteoblasts, thus coupling bone resorption to bone formation [29, 42]. A dampening of TGF-β signaling has been shown necessary for terminal osteoblast differentiation [43]. Furthermore, persistently high doses of TGF-β have been shown to inhibit osteoblast differentiation in a physiologically relevant manner as shown for osteogenesis imperfecta and other disorders of the ECM [19, 31, 44]. Transgenic overexpression of TGF-β2 in murine osteoblasts results in osteopenia, cortical thinning, and elevated osteocyte numbers [45]. Moreover, constitutive overexpression of Sp7/osterix in osteoblasts causes an accumulation of abnormal osteocytes in the absence of elevated osteoclast numbers [46]. Both mentioned mouse models phenotypically closely overlap with the *Gorab*^Prx1^ mutants. Interestingly, a direct upregulation of Sp7/osterix by TGF-β has been demonstrated [47]. Furthermore, the expression of *Dmp1*, which is strongly induced in *Gorab*-deficient bone tissues, was found to depend on *Tcf11*, which is also regulated by TGF-β [48, 49]. TGF-β has been demonstrated to promote Rankl-induced osteoclastogenesis [50]. In contrast to other models with elevated TGF-β signaling there is no consistent increase in osteoclast numbers in *Gorab*-deficient mice [19, 44]. This can be possibly attributed to the elevated Opg/Rankl ratio in our mouse model. One explanation for this might be the timing of exposure of osteoblast lineage cells to elevated TGF-β. In fibrillin 1-deficient mice TGF-β signaling is altered at the level of the stromal mesenchymal stem cell niche, while in *Gorab*^Prx1^ later stages of osteoblast differentiation are affected [44].

The localization of GORAB in the medial/trans Golgi is perfectly in line with an impairment of GAG chain elongation, which is carried out in this compartment [51]. The function of GORAB interaction partners ARF5, RAB6, and SCYL1 in retrograde Golgi trafficking suggests that the transport of factors relevant for glycanation might be impaired, similar to the regulation of EXT proteins by the Golgi protein GOLPH3 [3, 5, 52]. Golgi trafficking defects are often difficult to investigate *in vitro*. Even in the lethal phenotypes caused by mutations in *COG7* or *TRIP11*, encoding a component of the conserved oligomeric Golgi complex and the golgin Gmap-210, respectively, only relatively mild abnormalities were found in cultured mutant cells [53, 54]. Only after acute loss of Gmap-210 a global impairment of protein secretion and a retrograde trafficking delay became visible [55]. These results together with the clear glycanation impairment *in vivo* underline the significance of the proteoglycan alterations observed in GORAB deficient fibroblasts.

Although multiple lines of evidence point towards a central role of a perturbed proteoglycan-TGF-β axis in osteoblast lineage dysfunction in GO, it has been described that loss of Gorab impairs hedgehog signaling leading to reduced hair growth in *Gorab*^Null^ mutants [12]. Although GO patients show no hair phenotype and the bone phenotype is not typical for altered hedgehog signaling it is possible that this mechanism also contributes to the GO bone pathology [56]. Interestingly, mice deficient for the golgin Gmap-210 not only had a lung pathology very similar to *Gorab*^Null^ mutants, but were also reported to show cilia abnormalities [57]. On the other hand, also knockout of the Golgi enzyme gPapp impairing GAG sulfation leads to a lung phenotype closely resembling that of *Gorab*^Null^ mutants, which supports our hypothesis of a GAG-driven pathomechanism [58]. Further studies are needed to disentangle the contributions of these different pathways.

In summary, our study provides a link between the Golgi compartment, intracellular proteoglycan transport and glycanation, ECM disorganization and porosity, and TGF-β overactivation. Some aspects of this pathomechanism are also seen in chronological bone aging, but seem to occur already during postnatal growth in GO leading to the progeroid phenotype. The mechanism described here places GO in close proximity to congenital disorders of glycosylation with impaired proteoglycan synthesis.

## Methods

### Ethics statement

Permission for work with human cells was granted by the Charité Ethics Committee (approval number EA2/145/07). We or the referring clinicians obtained oral informed consent from patients for genetic testing and the use of fibroblasts derived from skin biopsies. Healthy control individuals also gave written informed consent for use of fibroblasts.

All animal experimental procedures were approved by the Landesamt für Gesundheitsschutz und Technische Sicherheit (LaGeTSi), Berlin, Germany (approval number G0213/12). Experiments using animal-derived materials were conducted according to the German law for animal protection (TierSchG).

### Animal procedures

All analyses were done on homozygous *Gorab*^Gt^ genetrap or *Gorab*^-/-^ (*Gorab*^Null^) embryos, or on female mice homozygous for the conditional *Gorab*^flox^ allele and heterozygous for the Prx1-cre transgene (*Gorab*^Prx1^). Homozygous conditional *Gorab*^flox^ mice without the cre allele from the same generation served as controls. All animals had been backcrossed with C57/Bl6 mice for at least 5 times.

### Generation of *Gorab*^Null^ mouse model

Gorab^*Null*^ animals were generated using genetrap ES-cell clone XG183 purchased from Bay Genomics, San Francisco, CA, USA. Germline deletion of *Gorab*^flox^ allele by crossing with CMV-cre mice resulted in homozygous mutants that were phenotypically identical to *Gorab*^Null^ mice.

### Construction of *Gorab*^flox^ conditional mouse model

The construction strategy was as shown in S1C Fig. A BAC clone containing the mouse *Gorab* locus, BAC clone bMQ-373H11 (129S7Ab2.2) was obtained from Geneservice Ltd (Cambridge, UK). A ~11.1kb region containing exons 2 to 4 of *Gorab* and the flanking introns was extracted into a pBluescript vector by recombination. LoxP sites were inserted into the targeting vector flanking exons 2 and 3 by homologous recombination. Gene targeting of mouse ES cells was done with the help from the Transgenic Core Facility of The University of Hong Kong. Correctly targeted clones were identified by Southern blot and injected into blastocysts from C57BL/6 mice to generate chimeric mice. Mice with germline transmission of the conditional cassette were crossed with ß-actin-flp mice to remove the neomycin selection cassette. The resulting animals were crossed with Prx1-cre mice to yield *Gorab*^Prx1^ mutants. Crossing with CMV-cre mice resulted in Gorab^-/-^ mutants through germline deletion.

### microCT analysis

microCT analysis for the *Gorab*^*Prx1*^ and *Gorab*^*Dmp1*^ mutants and corresponding littermate controls were done with Scanco μCT40 (Scanco Medical, Brüttisellen, Switzerland) at 10μm resolution; while *Gorab*^*Runx2*^ and littermate controls were analyzed with Skyscan 1172 (Bruker microCT, Luxemburg, Belgium) at 5μm resolution. Tibiae and vertebra were first fixed in 4% paraformaldehyde (PFA) in 4°C for 24 hours then scanned in 70% ethanol at 10μm resolution. The trabecular bone measurement was done for a region of 700 μm in the secondary spongiosa of proximal metaphysis of the tibia and the entire vertebral body. 1 mm of cortical bone was measured at a region 1200 μm below the proximal metaphysis of the tibia.

### Histology

E18.5 *Gorab*^Null^ embryos were sacrificed and dissected under PBS for lung biopsies to prevent collapse of the lung. Samples were then fixed in 4%PFA at 4°C for 24hours and then dehydrated through gradient ethanol from 70%, 80%, 90% and 100% ethanol at 4°C for 24hours at each step. The samples were then cleared in xylene twice at room temperature for 15min and then infiltrated with paraffin for 60 minutes three times followed by embedding. The embedded samples were sectioned and stained with hematoxylin/eosin for histology. Undecalcified mouse tibiae from E18.5 Gorab^Null^ embryos and four week old Gorab^Prx1^ mice were first fixed in 4% PFA and subsequently embedded in methylmethacrylate, MMA (Cat#00834, Polysciences, Eppelheim, Germany) and sectioned for histological studies. The fixed bone samples were dehydrated through gradient ethanol from 70%, 80%, 90% to 100% and twice in xylene for 24h in each step. The samples were then infiltrated with infiltration MMA (1%v/v polyethylene glycerol (Sigma-Aldrich, Munich, Germany) and 0.33% w/v benzoyl peroxide in MMA) for at least 24hours at 4°C. The polymerization was carried out at 4°C in polymerization solution (1%v/v polyethylene glycerol, 0.55% w/v benzoyl peroxide, 0.5% v/v N,N-dimethyl-p-toluidine in MMA). The embedded samples were then sectioned using a Leica RM2255 microtome (Leica, Wetzlar, Germany) at 5 μm thickness and subjected to Von Kossa/Van Giesson staining, Von Kossa/hematoxylin staining, Goldner trichrome staining or Pircosirius red staining.

### Histomorphometry

Histomorphometric analysis of the secondary spongiosa of proximal tibia of 4 weeks old GorabPrx1 mice was carried out using the software Osteomeasure (Osteometrics, Atlanta, USA). For mineral apposition rate determination, *Gorab*^Prx1^ and control animals were injected with calcein (10μl per gram body weight) at P23 and P26 and sacrificed at P28. The tibiae of the animals were fixed in 4% PFA and embedded in MMA as mentioned previously and then sectioned at 5μm thickness. The distance between the two lines of calcein labels at the mid bone shaft was subsequently imaged and measured for mineral apposition rate calculation.

### Transmission electron microscopy

All specimens were fixed for at least 2h at room temperature in 3% glutaraldehyde solution in 0.1M cacodylate buffer pH 7.4 and then cut into pieces of 1mm^3^. The samples were then washed in buffer and postfixed for 1h at 4°C in 1% osmium tetroxide, followed by dehydration through graded ethanol solutions and embedded in epoxy resin (glycidether 100). Semithin and ultrathin sections were cut with an ultramicrotome (Reichert Ultracut E). Ultrathin sections were treated with uranyl acetate and lead citrate, and examined with an electron microscope Philips EM 400.

### Quantitation of glycosaminoglycans (GAGs) in tissues

GAGs were prepared from E18.5 embryo skin, lung, cartilage and brain as described previously [59]. Each extract was digested with chondroitinase AC (Seikagaku Corp., Tokyo, Japan), chondroitinase B (IBEX Tech., Montreal, Canada), or heparinase (IBEX Tech., Montreal, Canada) for analyzing the disaccharide composition of CS, DS, or HS, respectively. Each digest was labeled with a fluorophore 2-aminobenzamide (2-AB) (Nacalai tesque, Kyoto, Japan), and analyzed by anion-exchange HPLC. To recover GAGs from bone specimens, tibia diaphysis from 4 week old *Gorab*^*Prx1*^ and wildtype mice were decalcified and digested with 20U of papain (Sigma-Aldrich, Milano, Italy) in 100mM sodium acetate, pH 5.6, 100mM EDTA and 5mM cysteine at 65°C for 48h. GAGs were purified by cetylpyridinium chloride precipitation and hyaluronic acid was removed by digestion with *Streptomyces* hyaluronidase (Seikagaku Corp., Tokyo, Japan) followed by ultrafiltration as described previously [60]. For CS and DS disaccharide composition, GAG aliquots were digested with chondroitinase ABC (AMSBIO, Abingdon, UK) (digesting chondroitin sulfate A, B and C) or chondroitinase ACII (Seikagaku Corp., Tokyo, Japan) (digesting chondroitin sulfate A and C). Each digest was labeled with 2-aminoacridone (Thermo Fisher Scientific, MA, USA) and analyzed by reverse phase HPLC as described previously [61]. The dermatan sulfate (chondroitin sulfate B) fraction was determined as disaccharide fraction undigestable by chondroitinase ACII vs. disaccharides digested by chondroitinase ABC.

### Immunoblotting and immunostaining

Cell lysates from in vitro cultures were extracted with RIPA buffer followed by sonication. Mouse tissue samples were pulverized in liquid nitrogen and lysates were extracted with 8M urea buffer, 1% SDS with sonication. For chondroitinase ABC digestion the buffer was exchanged to 0.1M Tris-HCl pH8.0 using microcon 10kDa centrifugal filter units and subsequently incubated at 37°C for 18 hours with or without chondroitinase ABC (ABCase, 0.3U/100μg protein). Mouse bone biopsies were first fixed in 4% PFA and then decalcified in Morses’ solution (10% Sodium Citrate, 20% Formic Acid). The decalcified bone were then embedded in paraffin and sectioned for immunostainings. Antibodies used are as follows: Anti-p-Smad2 (#3101, Cell Signaling, Leiden, The Netherlands), anti-SMAD2 (#3102, Cell Signaling, Leiden, The Netherlands), (Anti-Gapdh (#sc6215, Santa Cruz, Heidelberg, Germany), Anti-DCN (#AF143 and #AF1060, R&D, Abingdon, UK), Anti-BGN (#ENH020, Kerafast, Boston, USA), Anti-Sp7 (#ab22552, Abcam, Cambridge, UK), Anti-Spp1 (#MPIIIB10, DSHB, Iowa, USA), Anti-Dmp1 (#AF4386, R&D, Abingdon, UK). For immunofluorescence on cells we used the following antibodies: anti-decorin (#14667 Proteintech) anti-GM130 (clone 35, BD Biosciences), anti-VSV-tag (clone P5D4, Sigma). Sheep and rabbit GORAB antibodies were raised against GST-tagged N-terminal (1–130 aa) and C-terminal (301–369 aa) regions of human GORAB respectively and affinity purified against these same proteins. Serum was pre-cleared against GST before affinity purification on immunogen. Antibodies against TGN46 were provided by Dr S. Ponnambalam (University of Leeds, United Kingdom), while GD3A12 anti-DS antibody was a kind gift of Dr Toin van Kuppevelt (Radboud University Nijmegen Medical Center, The Netherlands)[26]. For immunohistochemistry, Vectastain elite ABC kit (Vector Laboratories, Burlingame, CA) was used for signal detection. For immunofluorescence on sections, the Tyramide signal amplification system (Perkin Elmer, Baesweiler, Germany) was used for signal development.

### *In vitro* differentiation of calvarial osteoblasts

Calvarial osteoblast progenitor cells were isolated as previously described [62]. Cells were seeded on 6-well plates in Alpha-MEM (Lonza, Basel, Switzerland) containing 10% fetal calf serum (FCS; Gibco, Life Technologies, Carlsbad, California, USA) as well as Pen/Strep (100U/mL, Lonza) and 2 mM ultra-glutamine (Lonza). Osteogenic differentiation was induced by with 50 μM L-ascorbate-2-phosphate and 10 mM beta-glycerophosphate.

### Alkaline phosphatase and matrix mineralization assays

Quantitative AP and matrix mineralization assays were performed as previously described [62]. Shortly, AP activity was determined by homogenizing 3 replicates separately in ALP-buffer1 (0.1 M Glycine; 1% NP-40; 1 mM MgCl2; 1 mM ZnCl2). After the addition of 1 volume of ALP-buffer2 (5 mM p-nitrophenyl phosphate [p-NPP], 0.1 M glycine, pH 9.6, 1 mM MgCl_2_, and 1 mM ZnCl_2_), reactions were incubated at 37°C for 30 min and stopped by addition of 1 M NaOH. The amount of p-NP released from the substrate p-NPP was recorded at 405 nm. AP activity is given as unit of absorption / μg protein / 30 min. Cells were fixed with 4% PFA prior to staining with Alizarin Red.

### *In vitro* TGF-ß reporter cell assay

Skin biopsies from E18.5 *Gorab*^Null^ embryos were homogenized in liquid nitrogen and soluble protein was extracted with 1X PBS, 1X complete protease inhibitor cocktail (Roche, Mannheim, Germany) for 16h at 4°C with agitation. Protein content of the lysates was then quantitated by BCA assay (Thermo Fisher, Dreieich, Germany) and 500μg of protein (either heat activated at 80°C for 10min for the total TGF-ß or without activation) was added to Plasminogen activator inhibitor-1–luciferase reporter mink lung epithelial cells (gift from Prof. Petra Knaus, Freie Universität, Berlin, Germany) and incubated for 16h. The lysates of the reporter cells were than collected for the luciferase activity using the Dual luciferase reporter system (Promega, Mannheim, Germany) and the amount of TGF-ß in skin lysates was quantitated by comparing with reporter cells treated with known amounts of recombinant TGF-ß (eBioscience, Frankfurt, Germany).

### Electrophoretic analysis of collagen secretion

Post-confluent human skin fibroblast were labelled for 18 h with 10μCi/ml L-[2,3,4,5-^3^H]-Proline in medium containing 0.15mM ascorbic acid. The secreted collagen in the medium was precipitated with 25% ammonium sulfate and then resuspended in 50mM Tris, 150mM NaCl pH7.5, which was then subjected to pepsin digestion (50μg/ml) at 4°C for 16h. The digested samples were then lyophilized and separated by SDS-PAGE and the labelled collagen chains were detected by fluorography.

### Analysis of cell-derived ECM

WT and GO human skin fibroblasts were seeded into 6-well plates and cultured for 8 days under standard conditions with media change on day 4. After 8 days cells were washed twice with PBS and the ECM was extracted by incubation with 300 μL of urea buffer (6 M urea, 25 mM dithiothretol (DTT) in 25 mM ammonium bicarbonate) for 10 min at RT. ECM samples were then subjected to SDS-PAGE and decorin was analyzed by WB.

### Analysis of decorin in MEFs

WT and GO MEFs were seeded into 6-well plates and cultured under standard conditions until they reached confluency. Cells were then stimulated to produce ECM components by incubation in fibroblast-specific serum-free medium (Lifeline Cell Technology) for 7 days. Cells were then lysed, samples were subjected to SDS-PAGE and decorin was analyzed by western blotting.

### Analysis of intra-Golgi decorin and GAG levels

WT and GO fibroblasts were seeded as a co-culture on glass coverslips to ensure fair comparison of signal intensities between controls and mutant cells and to exclude the influence of extrinsic factors. The cells were grown till 90% confluency. Cells were washed twice with PBS, fixed with 3% (wt/vol) PFA in PBS for 25 min at RT. Cells were then washed with PBS and the excess of paraformaldehyde was quenched with glycine. The cells were permeabilized by 4 min incubation in 0.1% (wt/vol) Triton X-100 in PBS. Cells were incubated with primary antibody diluted in PBS for 1 hour at RT and incubated three times with PBS for 5 min. Then coverslips were incubated for 1 h with secondary antibody diluted in PBS and incubated three times with PBS for 5 min and twice in ddH_2_0 for 5 min. In case of the GD3A12 antibody detecting dermatan sulfate, the primary antibody was recognized by P5D4 clonal antibody against VSV-tag prior to incubation with fluorescently-conjugated secondary antibody. Coverslips were dried before mounting in Mowiol 4–88 and images were acquired on a Ti inverted microscope (Nikon) using a x60/1.4 Plan Apo objective, Proscan II motorized stage (Prior Scientific) and R6 CCD camera (QImaging). A SpectraX LED light engine (Lumencore), quad dichroic (Semrock) and motorized emission filter wheel (Prior Scientific) with single bandpass filters for FITC, TRITC and Cy5 (Semrock) were used to collect image sequences at each position in the tile. Images were acquired and then aligned and stitched using NIS Elements software (Nikon). These stitched images were then exported as a single TIFF image for further processing in Fiji software [63]. The amount of intra-Golgi decorin and GAG was measured by comparing fluorescence intensity levels with reference to the Golgi markers GM130 and TGN46. GORAB staining was employed to discriminate between WT and GO fibroblasts.

### Gene expression analysis

#### RNA isolation from cultured cells

Total RNA was isolated from cultured skin fibroblasts or from osteoblast cultures at time 0 (i.er., without stimulation), 3 days, 6 days, and 12 days using TRIzol reagent (Life Technologies, Darmstadt, Germany). RNA integrity was confirmed using the RNA 6000 Nano Kit on the 2100 Bioanalyzer (Agilent Technologies, Santa Clara, USA).

#### RNA isolation from cortical bone tissue

For isolation of femoral cortical bone epiphyses were cut off, and bone marrow was flushed out. The hollow bone shaft was then digested twice with 0.2% collagenase (Sigma-Aldrich, Munich, Germany) in isolation buffer (70mM NaCl, 10mM NaHCO_3_, 60mM sorbitol, 30mM KCl, 3mM K_2_HPO_4_, 1mM CaCl_2_, 0.1% bovine serum albumin, 0.5% glucose, 25 mM HEPES) at 37°C for 20 minutes each under vigorous shaking. The cleared bone shaft was frozen in liquid nitrogen and then homogenized with pestle and mortar. RNA was extracted from the homogenized tissue with TRIzol reagent (Life Technologies, Darmstadt, Germany).

#### Microarray expression analysis

Femoral and tibial cortical bone mRNA from 4 week old control and *Gorab*^Prx1^ mutants (N = 3) was hybridized on an MG ST 1.0 microarray (Affymetrix, High Wycombe, United Kingdom). The raw image files were converted to cel files using the dChip software. The data were subjected to a quality assurance analysis using the simpleaffy package of Bioconductor. Normalization and summarization of the probe set data were conducted using the gcrma package.

#### RNA-sequencing and analysis

For library preparation the TruSeq RNA Sample Prep Kit was used and after cluster generation by the TruSeq PE Cluster Kit 75bp paired-end sequencing occurred on an HiSeq 2000 machine (Illumina, San Diego, USA). We analyzed the differential gene expression of the RNA-seq data at the different time points by the DESeq2 package, where the raw count of the reads is normalized taking into account the sample size factor and for further sources of technical biases such as differing dependence on GC content or the gene length. For the comparison of the aligned reads across samples, we used Bioconductor package DESeq2 which uses a generalized linear model (GLM) of the negative binomial distribution of the normalized gene counts.

#### Quantitative PCR

RNA was reverse-transcribed using the RevertAid H Minus First Strand cDNA Synthesis Kit (Life Technologies, Darmstadt, Germany) with random hexamer primers. Quantitative PCR was performed with SYBR green (Life Technologies, Darmstadt, Germany) on ABI Prism 7500 (Life Technologies, Darmstadt, Germany). Expression levels were normalized to Gapdh expression. Primer sequences are given in S2 Table.

### Statistical analysis

All values are shown as mean ± SD. Data were analyzed with unpaired-two-tailed Student’s t test. Microarray data was analyzed by One-Way-ANOVA. A p-value smaller than 0.05 was considered statistically significant. In all figures, * p-value < 0.05, ** p-value < 0.01, *** p-value < 0.001, n.s. = not significant

## Supporting information

S1 Fig Animal models used in this study. (A) Schematic illustration of the position of the genetrap cassette (β-geo) within intron 1 of the *Gorab* locus in XG183 *Gorab*^Null^ mouse. (B) Quantitative PCR showing successful inactivation of *Gorab* in *Gorab*^Null^ mouse skin (N = 3). (C) Strategy for generation of *Gorab*^flox^ conditional mice. (D) Detection of targeted *Gorab* allele by Southern blot.
(PDF)

S2 Fig Characterization of lethal *Gorab*^Null^ mice. (A) Pictures of a newborn (P0) *Gorab*^Null^ mouse which died shortly after birth illustrating no significant morphological changes and no cutis laxa phenotype. (B) Hematoxylin/eosine stained skin section from P0 *Gorab*^*Null*^ mouse mutant and control showing no significant alteration. Scale bar = 50μm. (C) Lung sections from P0 *Gorab*^*Null*^ mutant and control after breathing stained by hematoxylin/eosine showing collapsed alveoli and reduced septation. (D) Lung sections from E18.5 *Gorab*^*Null*^ and control embryo before breathing stained by hematoxylin/eosine or toluidine, also showing reduced airspace thus underlining that the phenotype is not due to an inability for respiratory excursions. Scale bar = 200μm.
(PDF)

S3 Fig Characterization of bone phenotype in *Gorab*^Null^ mouse embryos. (A) Alizarin red/ alcian blue staining of E18.5 *Gorab*^Null^ skeleton showing no observable difference in the rib cage and forelimb, but a slightly smaller mandibula. (B) μCT reconstructed E18.5 *Gorab*^Null^ mouse skull showing enlarged fontanels. (C) μCT reconstructed image of the tibia midshaft of E18.5 *Gorab*^*Null*^ embryo in axial orientation. Apart from a slightly shorter diameter there is no significant difference in the developing, still highly porous cortical bone between mutant and control. (D) Representative sections of proximal tibia from E18.5 control and *Gorab*^Null^ animals stained with Goldner trichrome/ von Kossa. Scale bar = 200μm. (E) Histomorphometric analysis of E18.5 control and *Gorab*^Null^ tibia trabecular bone (N = 3). (F) Expression of osteocyte markers in bone of P0 *Gorab*^*Null*^ mutants (N = 3) already showing upregulation of *Dmp1* and downregulation of *Sost*, indicating a delay in osteocyte differentiation. (G) Alkaline phosphatase (AP) and alizarin red staining after 7 and 21 days of osteogenic differentiation of primary calvarial osteoblasts from E18.5 *Gorab*^*Null*^ mutants, respectively. (H) Alkaline phosphatase (AP) enzymatic activity of primary calvarial osteoblast of E18.5 *Gorab*^*Null*^ comparing to control after 4 days (Control vs. *Gorab*^*Null*^, N = 11 vs. 5) and 7 days Control vs *Gorab*^*Null*^, N = 13 vs. 6) of osteogenic differentiation.
(PDF)

S4 Fig Gorab expression during normal osteoblast differentiation and its loss in the different conditional mouse models. (A) Expression levels of *Gorab* in comparison to several transcription factors and ECM proteins in differentiating calvarial osteoblasts from three independent experiments with four calvariae each. Note peak of *Gorab* expression at day 6 of differentiation together with *Col1a1* and *Dcn* while the late osteoblast marker *Dmp1* is only significantly expressed at day 12. *Prrx1* (encoding Prx1) and *Runx2* expression are high at the beginning of osteogenic differentiation at day 0. (B) qPCR analysis of *Gorab* expression in tibia diaphysis of 12 week old control (N = 3), *Gorab*^*Prx1*^ (N = 3), *Gorab*^*Runx2*^ (N = 3) and *Gorab*^*Dmp1*^ (N = 3) mice demonstrating similar efficiencies for cre-induced inactivation.
(PDF)

S5 Fig microCT analysis of different Gorab mutants. microCT analysis of trabecular bone volume fraction (BV/TV), trabecular number (Tb.N), trabecular thickness (Tb.Th) and trabecular separation (Tb.Sp) of (A) tibia and (B) sixth lumbar vertebrae of twelve week old *Gorab*^Prx1^ (N = 15), *Gorab*^Runx2^ (N = 5), *Gorab*^Dmp1^ (N = 8) and corresponding littermate control animals (N = 14, N = 5, N = 9 respectively).
(PDF)

S6 Fig Histomorphometric analysis of a bone biopsy from a GO patient. (A) Von Kossa/Van Gieson staining and (B) Goldner trichrome staining of bone biopsy from a nine year old GO patient. (C) Histomorphometric analysis showing strong reduction of trabecular bone volume fraction (BV/TV), accumulation of osteoid (OV/TV and OS/BS) and increase in osteoblast (N.Ob/B.Pm) and osteocyte number (N.Ot/trab B.Pm and N.Ot/cort B.Pm) in the GO patient similar to *Gorab*^*Prx1*^ mice.
(PDF)

S7 Fig Characterization of collagen secretion from skin fibroblasts from GO patients and GAG content in E18.5 *Gorab*^Null^ mice skin. (A) Electrophoresis of ^3^H labeled collagen from control and GO fibroblast culture (N = 3). Note comparable secretion levels. (B) Pulse-chase experiment for global protein secretion by cultured control and GO fibroblasts (N = 3) showing no significant difference. (C) Quantitation of heparan sulfate and hyaluronan in skin, cartilage, lung and brain of E18.5 *Gorab*^Null^ mice (N = 3–4). (D) Immunoblot of biglycan in lysates from mouse embryonic fibroblasts. Note migration of glycanated band around 100 kDa and of the core protein at around 45 kDa. Mutant cells show a stronger core protein band and a smear of incompletely glycanated protein species. (E, F) Immunoblot of decorin (E) and biglycan (F) in skin lysates from E18.5 *Gorab*^Null^ mice with and without chondroitinase ABC digestion (ABC). Note increased detection of the core protein after chondroitinase ABC digestion. The fully glycanated bands are not clearly detected, probably as a consequence of the special sample preparation necessary for the enzyme incubation. (G) qPCR analysis of biglycan and decorin expression in skin of E18.5 *Gorab*^*Null*^ embryo (N = 4) and (H) femur diaphysis from four week old *Gorab*^*Prx1*^ mice (N = 6).
(PDF)

S1 Table Expression profiling in cortical bone from four week old *Gorab*^Prx1^ mutants and controls. (XLSX)

S2 Table Primers used for quantitative PCR analysis. (XLSX)
