# Supplementary figures and images for: Impaired proteoglycan glycosylation, elevated TGF-β signaling, and abnormal osteoblast differentiation as the basis for bone fragility in a mouse model for gerodermia osteodysplastica
Source: PLoS Genet. 2018 Mar 21;14(3):e1007242. doi: 10.1371/journal.pgen.1007242 (PMC5880397; doi:10.1371/journal.pgen.1007242)

# S1 Figure

**A**

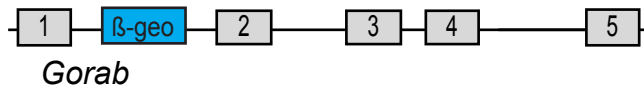

**B**

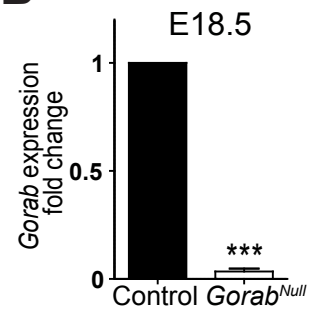

**C**

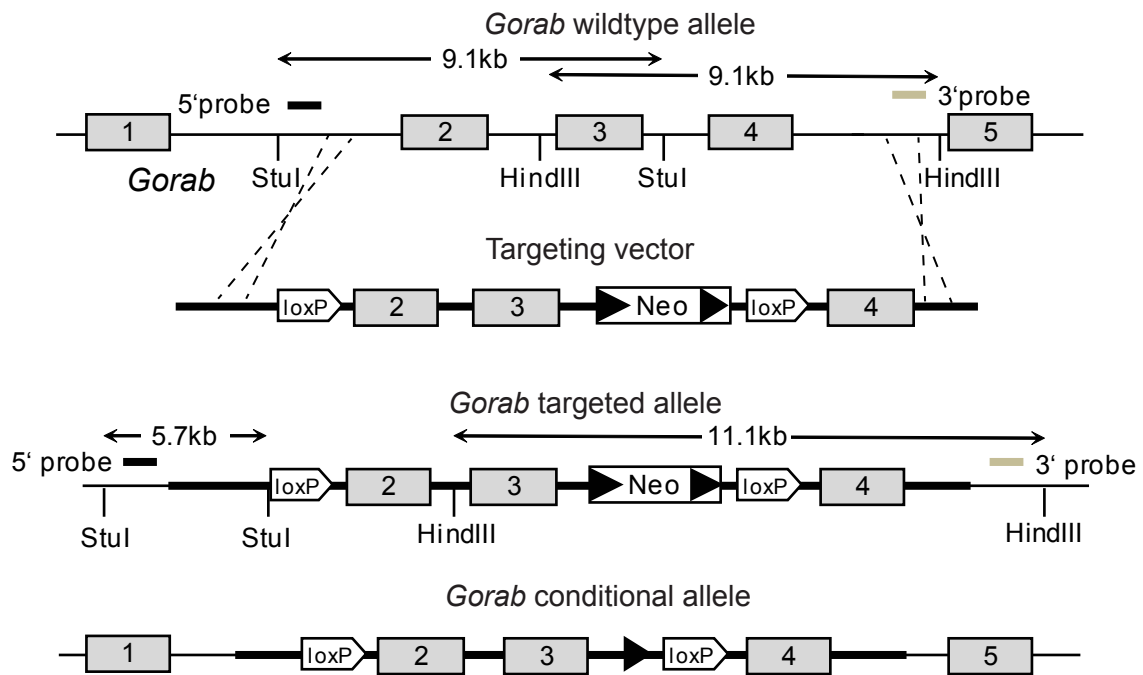

**D**

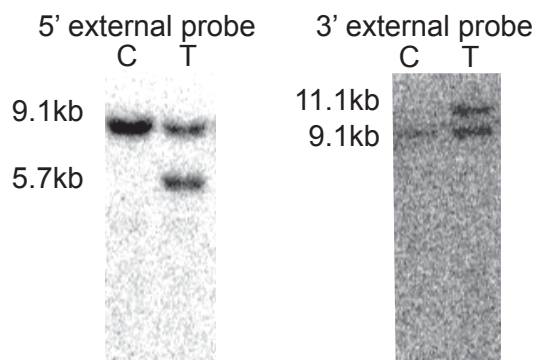

Supplement: S1 Fig — (A) Schematic illustration of the position of the genetrap cassette (β-geo) within intron 1 of the Gorab locus in XG183 GorabNull mouse. (B) Quantitative PCR showing successful inactivation of Gorab in GorabNull mouse skin (N = 3). (C) Strategy for generation of Gorabflox conditional mice. (D) Detection of targeted Gorab allele by Southern blot. (PDF) [file pgen.1007242.s001.pdf]

## S2 Figure

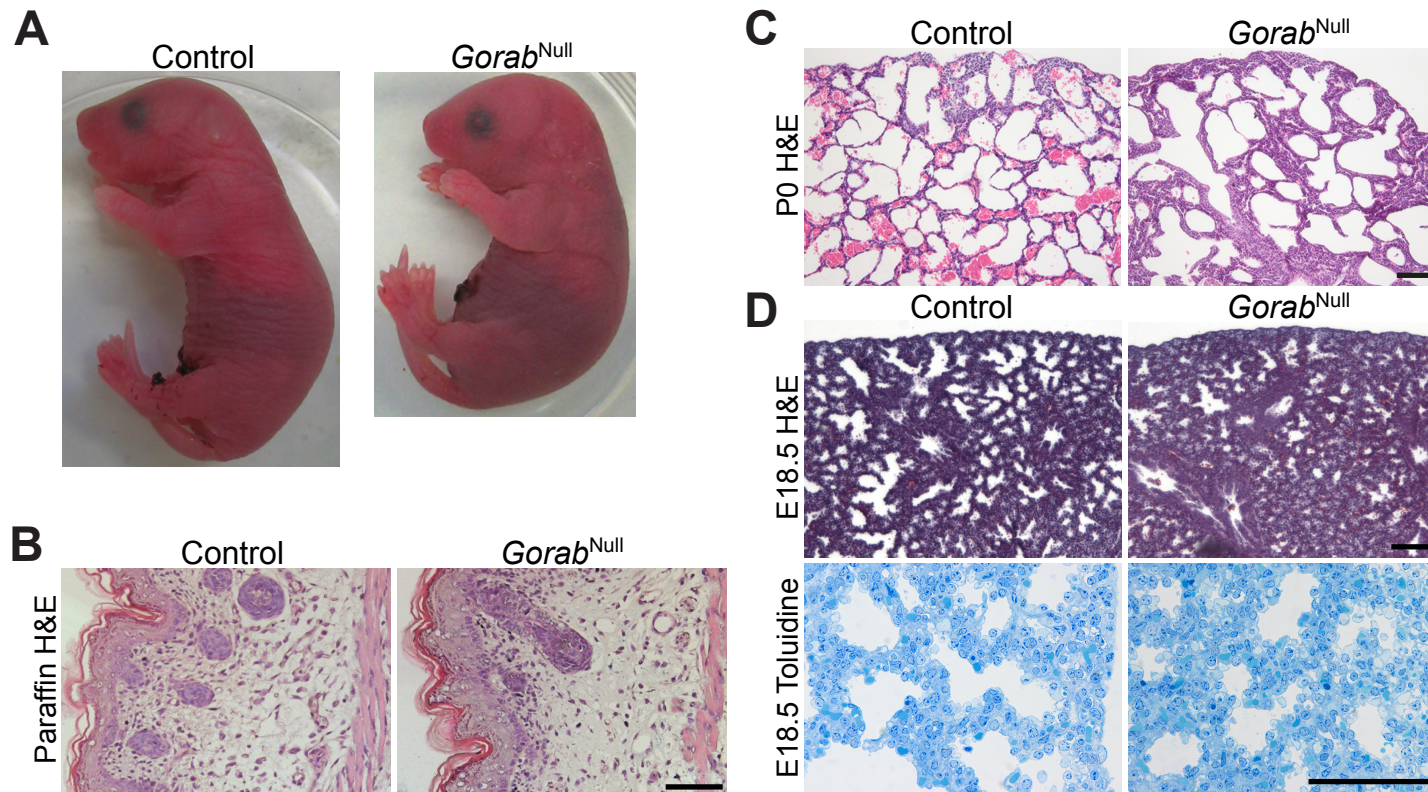

Supplement: S2 Fig — (A) Pictures of a newborn (P0) GorabNull mouse which died shortly after birth illustrating no significant morphological changes and no cutis laxa phenotype. (B) Hematoxylin/eosine stained skin section from P0 GorabNull mouse mutant and control showing no significant alteration. Scale bar = 50μm. (C) Lung sections from P0 GorabNull mutant and control after breathing stained by hematoxylin/eosine showing collapsed alveoli and reduced septation. (D) Lung sections from E18.5 GorabNull and control embryo before breathing stained by hematoxylin/eosine or toluidine, also showing reduced airspace thus underlining that the phenotype is not due to an inability for respiratory excursions. Scale bar = 200μm. (PDF) [file pgen.1007242.s002.pdf]

# S3 Figure

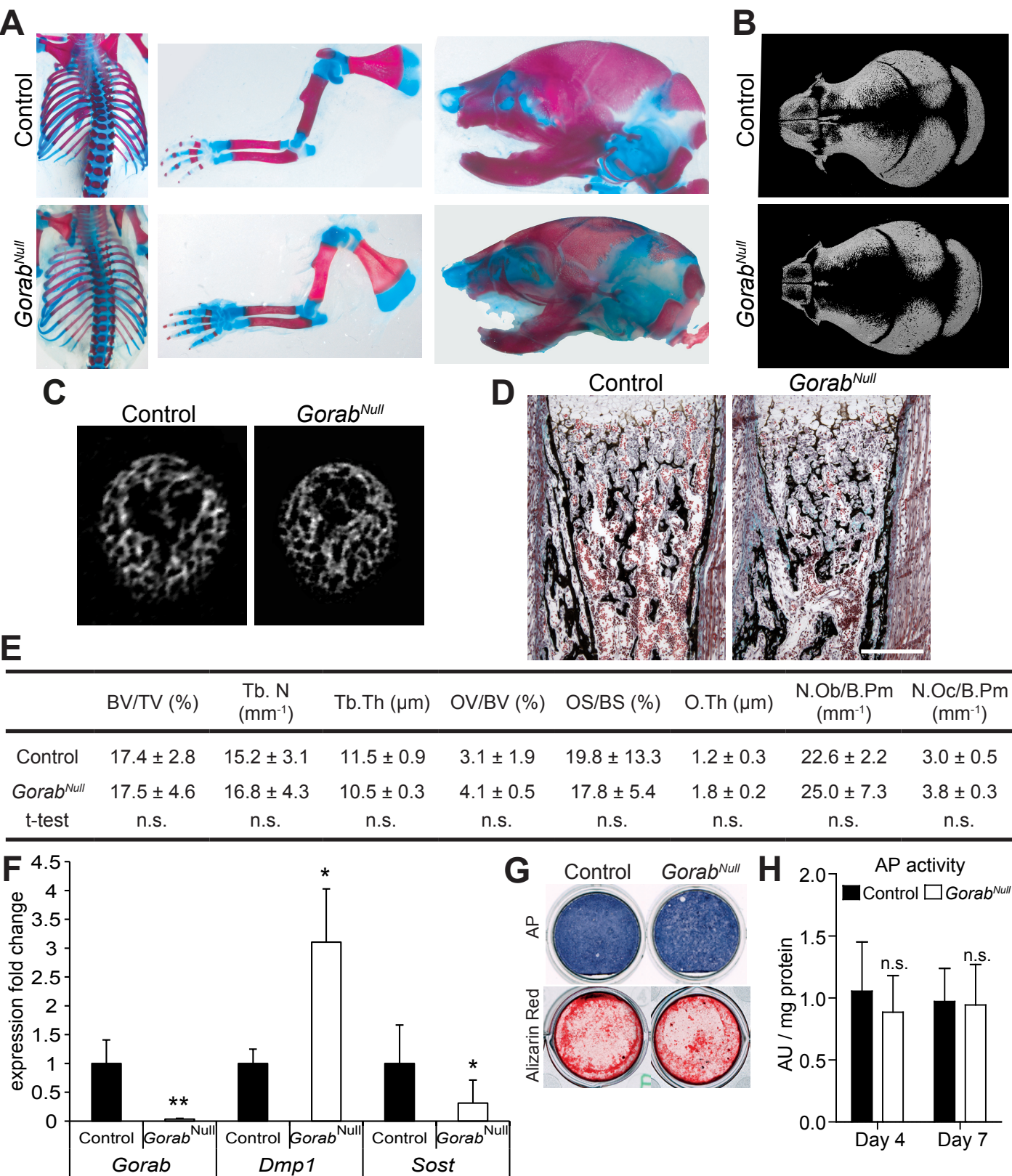

Supplement: S3 Fig — (A) Alizarin red/ alcian blue staining of E18.5 GorabNull skeleton showing no observable difference in the rib cage and forelimb, but a slightly smaller mandibula. (B) μCT reconstructed E18.5 GorabNull mouse skull showing enlarged fontanels. (C) μCT reconstructed image of the tibia midshaft of E18.5 GorabNull embryo in axial orientation. Apart from a slightly shorter diameter there is no significant difference in the developing, still highly porous cortical bone between mutant and control. (D) Representative sections of proximal tibia from E18.5 control and GorabNull animals stained with Goldner trichrome/ von Kossa. Scale bar = 200μm. (E) Histomorphometric analysis of E18.5 control and GorabNull tibia trabecular bone (N = 3). (F) Expression of osteocyte markers in bone of P0 GorabNull mutants (N = 3) already showing upregulation of Dmp1 and downregulation of Sost, indicating a delay in osteocyte differentiation. (G) Alkaline phosphatase (AP) and alizarin red staining after 7 and 21 days of osteogenic differentiation of primary calvarial osteoblasts from E18.5 GorabNull mutants, respectively. (H) Alkaline phosphatase (AP) enzymatic activity of primary calvarial osteoblast of E18.5 GorabNull comparing to control after 4 days (Control vs. GorabNull, N = 11 vs. 5) and 7 days Control vs GorabNull, N = 13 vs. 6) of osteogenic differentiation. (PDF) [file pgen.1007242.s003.pdf]

## S4 Figure

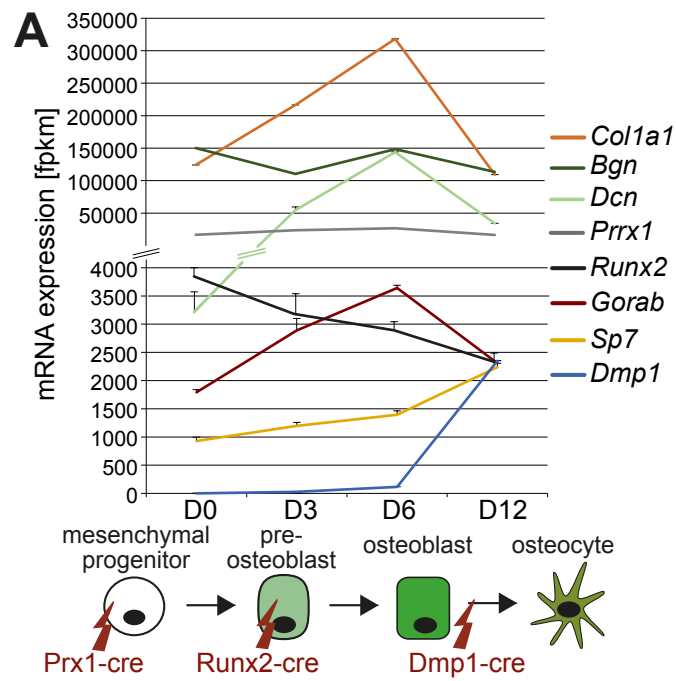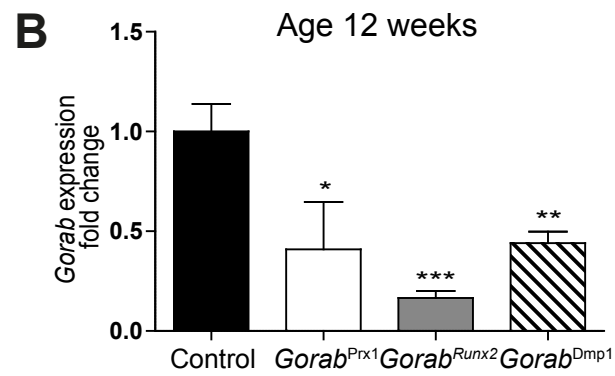

Supplement: S4 Fig — (A) Expression levels of Gorab in comparison to several transcription factors and ECM proteins in differentiating calvarial osteoblasts from three independent experiments with four calvariae each. Note peak of Gorab expression at day 6 of differentiation together with Col1a1 and Dcn while the late osteoblast marker Dmp1 is only significantly expressed at day 12. Prrx1 (encoding Prx1) and Runx2 expression are high at the beginning of osteogenic differentiation at day 0. (B) qPCR analysis of Gorab expression in tibia diaphysis of 12 week old control (N = 3), GorabPrx1 (N = 3), GorabRunx2 (N = 3) and GorabDmp1 (N = 3) mice demonstrating similar efficiencies for cre-induced inactivation. (PDF) [file pgen.1007242.s004.pdf]

# S5 Figure

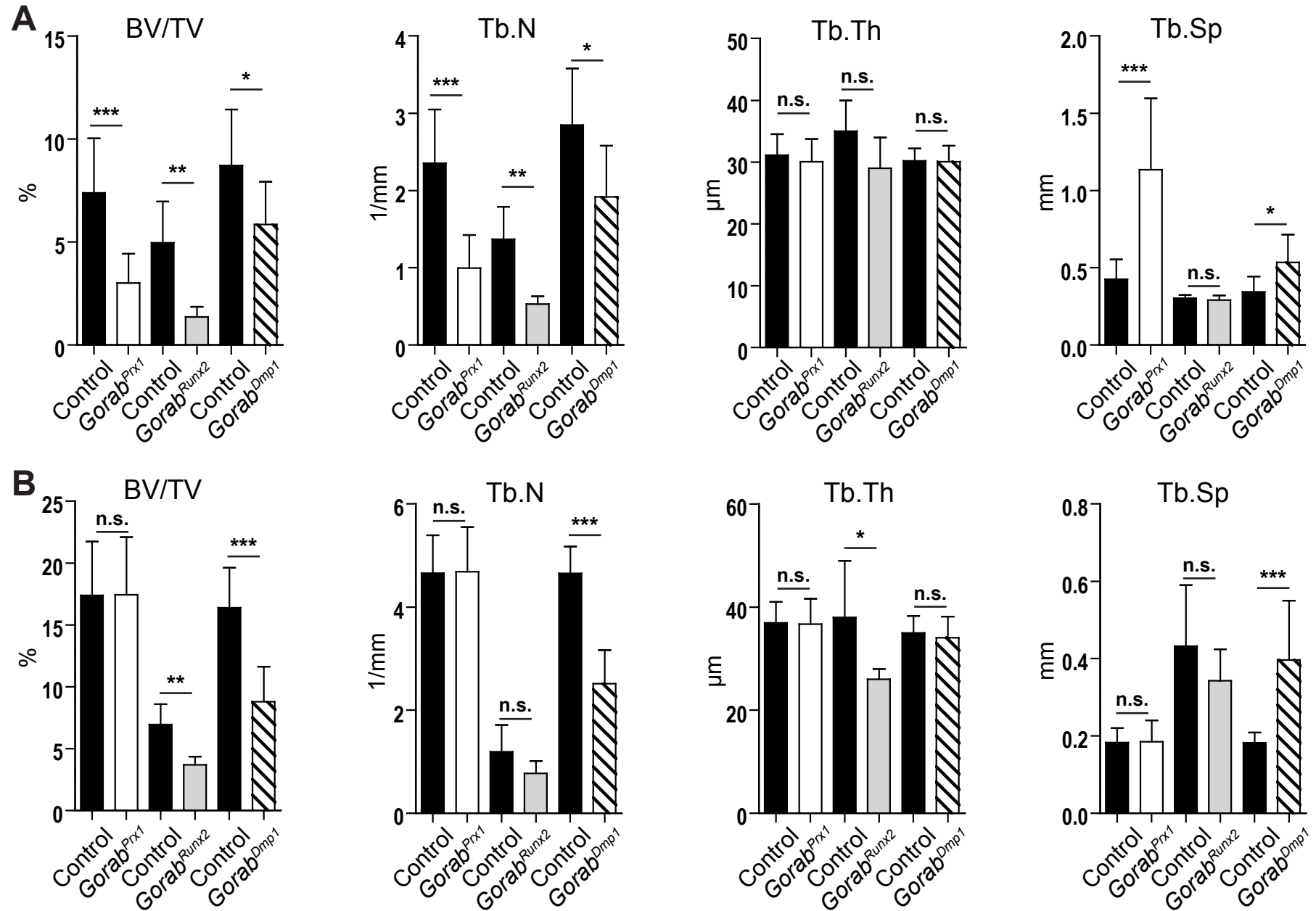

Supplement: S5 Fig — microCT analysis of trabecular bone volume fraction (BV/TV), trabecular number (Tb.N), trabecular thickness (Tb.Th) and trabecular separation (Tb.Sp) of (A) tibia and (B) sixth lumbar vertebrae of twelve week old GorabPrx1 (N = 15), GorabRunx2 (N = 5), GorabDmp1 (N = 8) and corresponding littermate control animals (N = 14, N = 5, N = 9 respectively). (PDF) [file pgen.1007242.s005.pdf]

# S7 Figure

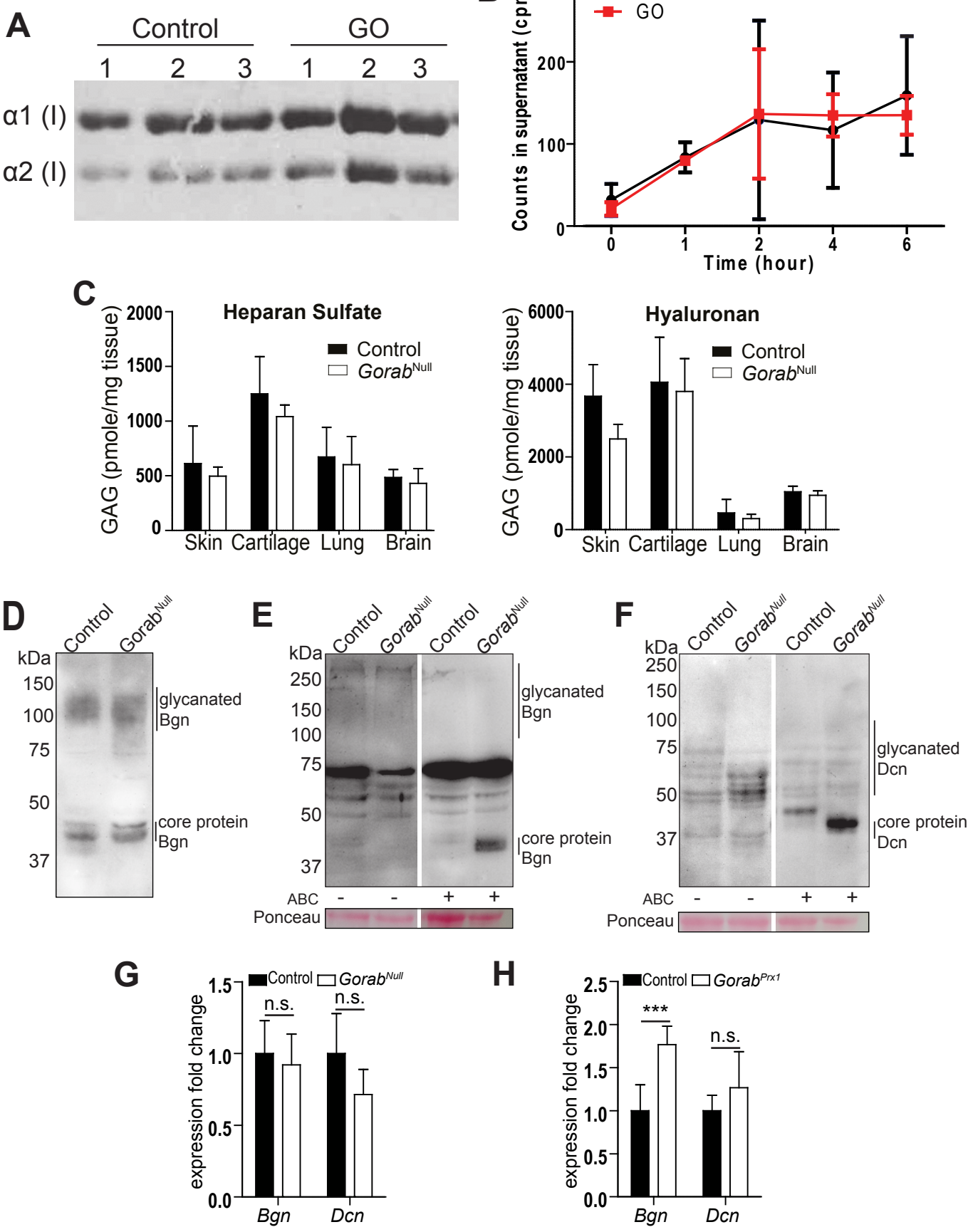

Supplement: S7 Fig — (A) Electrophoresis of 3H labeled collagen from control and GO fibroblast culture (N = 3). Note comparable secretion levels. (B) Pulse-chase experiment for global protein secretion by cultured control and GO fibroblasts (N = 3) showing no significant difference. (C) Quantitation of heparan sulfate and hyaluronan in skin, cartilage, lung and brain of E18.5 GorabNull mice (N = 3–4). (D) Immunoblot of biglycan in lysates from mouse embryonic fibroblasts. Note migration of glycanated band around 100 kDa and of the core protein at around 45 kDa. Mutant cells show a stronger core protein band and a smear of incompletely glycanated protein species. (E, F) Immunoblot of decorin (E) and biglycan (F) in skin lysates from E18.5 GorabNull mice with and without chondroitinase ABC digestion (ABC). Note increased detection of the core protein after chondroitinase ABC digestion. The fully glycanated bands are not clearly detected, probably as a consequence of the special sample preparation necessary for the enzyme incubation. (G) qPCR analysis of biglycan and decorin expression in skin of E18.5 GorabNull embryo (N = 4) and (H) femur diaphysis from four week old GorabPrx1 mice (N = 6). (PDF) [file pgen.1007242.s007.pdf]
