# Supplementary material for: Impaired proteoglycan glycosylation, elevated TGF-β signaling, and abnormal osteoblast differentiation as the basis for bone fragility in a mouse model for gerodermia osteodysplastica
Source: PLoS Genet. 2018 Mar 21;14(3):e1007242. doi: 10.1371/journal.pgen.1007242 (PMC5880397; doi:10.1371/journal.pgen.1007242)
Supplement: S6 Fig — (A) Von Kossa/Van Gieson staining and (B) Goldner trichrome staining of bone biopsy from a nine year old GO patient. (C) Histomorphometric analysis showing strong reduction of trabecular bone volume fraction (BV/TV), accumulation of osteoid (OV/TV and OS/BS) and increase in osteoblast (N.Ob/B.Pm) and osteocyte number (N.Ot/trab B.Pm and N.Ot/cort B.Pm) in the GO patient similar to GorabPrx1 mice. (PDF) [file pgen.1007242.s006.pdf]

S6 Figure

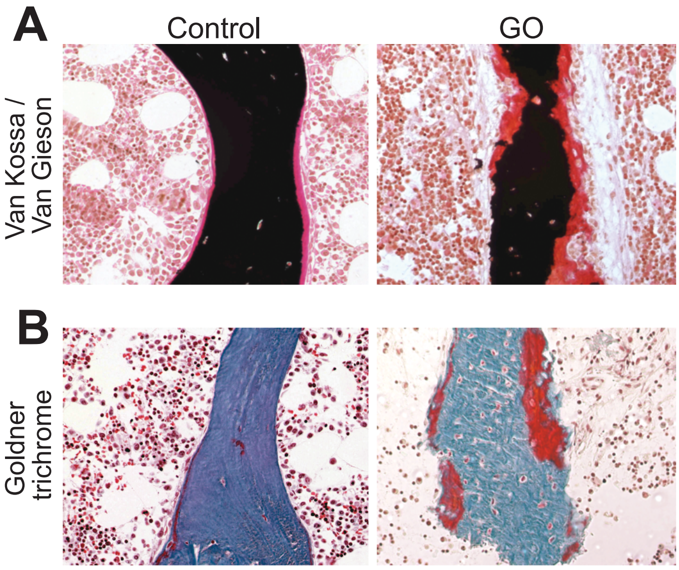

C

	Control	GO
BV/TV (%)	25.74	7.86
Tb.Th (µm)	154.06	51.2
Tb.N (mm^{-1})	1.67	1.54
OV/TV (%)	3.15	8.66
OS/BS (%)	32.69	34.95
O.Th (µm)	4.97	12.96
N.Ob/B.Pm (mm^{-1})	6.46	13.57
N.Ot/trab B.Pm (mm^{-1})	10.38	23.34
N.Ot/cort B.Pm (mm^{-1})	55.96	81.72
N.Oc/B.Pm (mm^{-1})	0.76	1.2
